# A review on potential heterocycles for the treatment of glioblastoma targeting receptor tyrosine kinases

**DOI:** 10.32604/or.2024.047042

**Published:** 2024-04-23

**Authors:** NILAM BHUSARE, MAUSHMI KUMAR

**Affiliations:** Somaiya Institute for Research & Consultancy, Somaiya Vidyavihar University, Vidyavihar (East), Mumbai, 400077, India

**Keywords:** Glioblastoma, Kinase pathway, Pyrimidine, Quinazoline, Heterocycles

## Abstract

Glioblastoma, the most aggressive form of brain tumor, poses significant challenges in terms of treatment success and patient survival. Current treatment modalities for glioblastoma include radiation therapy, surgical intervention, and chemotherapy. Unfortunately, the median survival rate remains dishearteningly low at 12–15 months. One of the major obstacles in treating glioblastoma is the recurrence of tumors, making chemotherapy the primary approach for secondary glioma patients. However, the efficacy of drugs is hampered by the presence of the blood-brain barrier and multidrug resistance mechanisms. Consequently, considerable research efforts have been directed toward understanding the underlying signaling pathways involved in glioma and developing targeted drugs. To tackle glioma, numerous studies have examined kinase-downstream signaling pathways such as RAS-RAF-MEK-ERK-MPAK. By targeting specific signaling pathways, heterocyclic compounds have demonstrated efficacy in glioma therapeutics. Additionally, key kinases including phosphatidylinositol 3-kinase (PI3K), serine/threonine kinase, cytoplasmic tyrosine kinase (CTK), receptor tyrosine kinase (RTK) and lipid kinase (LK) have been considered for investigation. These pathways play crucial roles in drug effectiveness in glioma treatment. Heterocyclic compounds, encompassing pyrimidine, thiazole, quinazoline, imidazole, indole, acridone, triazine, and other derivatives, have shown promising results in targeting these pathways. As part of this review, we propose exploring novel structures with low toxicity and high potency for glioma treatment. The development of these compounds should strive to overcome multidrug resistance mechanisms and efficiently penetrate the blood-brain barrier. By optimizing the chemical properties and designing compounds with enhanced drug-like characteristics, we can maximize their therapeutic value and minimize adverse effects. Considering the complex nature of glioblastoma, these novel structures should be rigorously tested and evaluated for their efficacy and safety profiles.

## Introduction

Cancer remains a formidable global health challenge, claiming the lives of millions of individuals worldwide [1]. The major types of prevalent cancers are carcinoma, sarcoma, leukemia and lymphoma [2]. The specific organ involved in cancer results in different types of cancer, such as kidney [3], bladder [4], lung [5], breast [6], colon or colorectal [7] and brain cancer [8]. Among all cancers, leukemia, a type of cancer that affects the blood and bone marrow, is the most commonly diagnosed malignancy in children, closely followed by brain cancer, accounting for up to 25% of cases [9]. Brain tumors can range from benign to malignant and can potentially metastasize. According to research, the effects of brain tumors can vary depending on the specific area of the brain that is affected. For instance, if the tumor develops in the temporal lobe, the patient may experience difficulties with hearing and visualization. Conversely, if the tumor affects the frontal lobe, it can lead to changes in the patient’s personality due to disruptions in their mental abilities. In more severe cases, brain tumors can even result in issues such as incontinence and impairments in gait [10]. Visualizing brain tumors plays a crucial role in diagnosing the stage and precise location of the tumor. This can be achieved through both invasive and non-invasive imaging techniques. Invasive procedures, such as catheter angiography, are commonly used to examine the tumor’s blood vessels and surrounding structures. However, non-invasive techniques like computed tomography (CT) and magnetic resonance imaging (MRI) have become invaluable in providing detailed and accurate images of brain lesions. The gold standard imaging technique has been used to scan tumors because it better visualizes the complexity and heterogeneity of tumor lesions [11].

**Figure 1:**
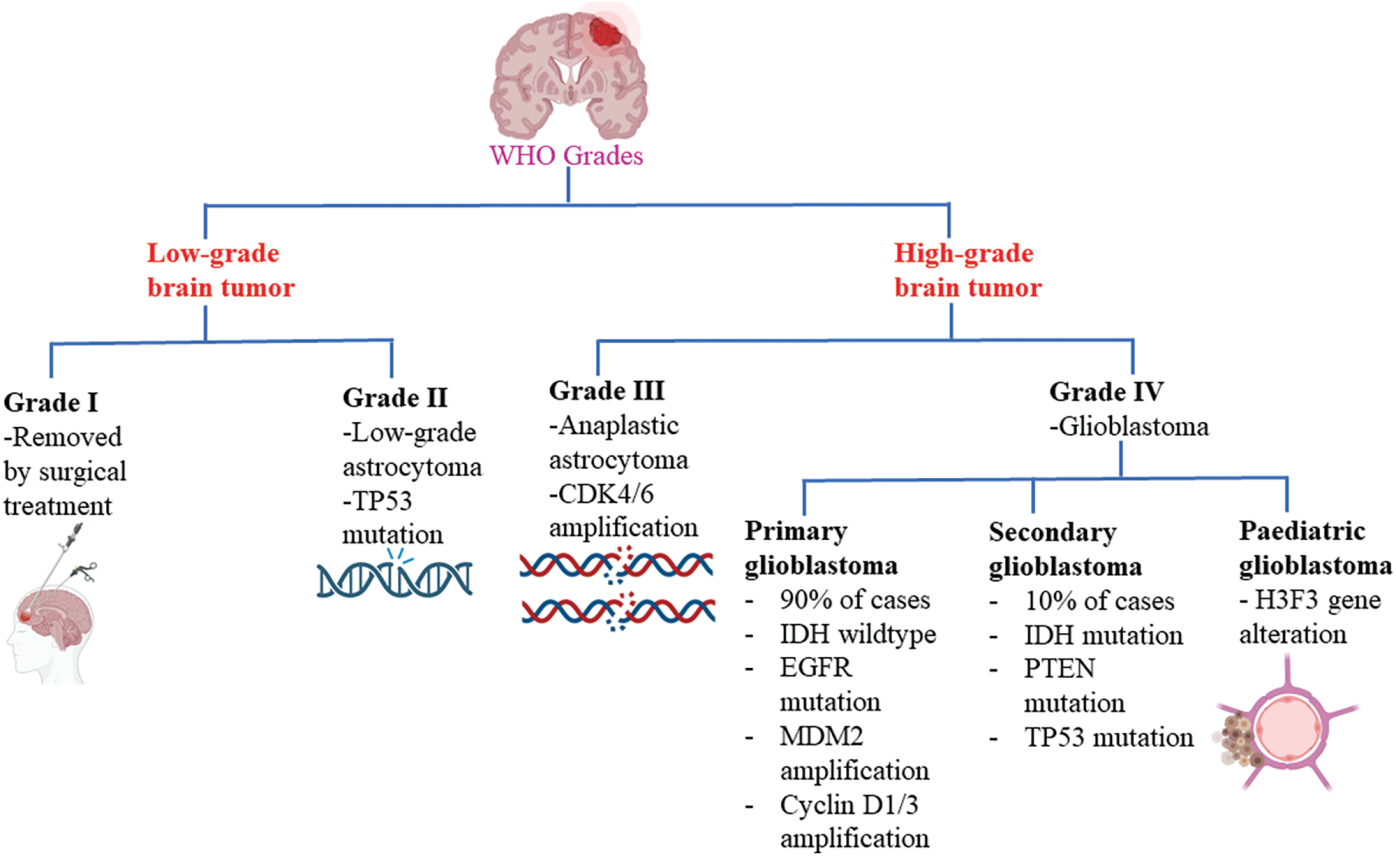
Glioblastoma WHO grades. CDK4/6: Cyclin-dependent kinase-4/6; IDH: Isocitrate dehydrogenase; EGFR: Epidermal growth factor receptor; MDM2: Mouse double minute 2; PTEN: Phosphatase and tensin homolog.

Brain tumors are classified based on the specific types of cells from which they originate. Gliomas, for instance, originate from glial cells, while meningiomas are abnormal growths of the meninges. Ependymomas are associated with ependymocyte cells that line the CSF-filled ventricles and astrocytomas develop from astrocytes, which are star-shaped glial cells, among others [12]. Among all brain tumors, one of the most challenging and resistant to treatment is glioblastoma. Glioblastomas account for 51% of initial malignant brain tumors, making them a significant concern in healthcare. With an annual occurrence of 3.0 to 3.6 instances per 100,000 people or approximately 240,000 new cases globally, glioblastoma is the most prevalent brain tumor in adults [13]. Glioblastoma multiforme (GBM) is a pernicious form of astrocytoma and affects more than 60% of brain tumor patients in adulthood [14]. To further characterize gliomas, they are also graded according to their cancer stages, ranging from the least offensive to a drastic terminal illness [15]. The World Health Organization (WHO) has established a grading system for gliomas, categorizing them into grades I to IV based on their potential for severe complications (Fig. 1). Low-grade tumors (grades I and II) have a relatively better prognosis compared to high-grade tumors (grades III and IV), which are malignant and often associated with serious complications [16]. The low proliferative power of lesions is related to grade I gliomas, which can be treated surgically. On the other hand, grade IV gliomas, also known as glioblastomas, are highly invasive and aggressive. GBM is predominantly observed in individuals aged 55 to 60 years and falls under the category of grade IV gliomas [10,17]. In addition to these classifications, glioblastomas can also be categorized as primary or secondary. Primary GBMs emerge *de novo* without any prior clinical evidence, whereas secondary GBMs develop from pre-existing low-grade GBMs [18] Primary GBMs are indicated by the overexpression, mutation, or multiplication of specific genes such as mouse double minute 2 (MDM2) and epidermal growth factor receptor (EGFR), as well as loss of heterozygosity. Secondary GBMs, on the other hand, exhibit distinctive molecular features such as platelet-derived growth factor receptor alpha (PDGFR-α) overexpression, mutation of isocitrate dehydrogenase (IDH)1/2, TP53 and ATRX. Furthermore, a third significant class of pediatric GBMs has emerged based on alterations in the histone gene H3F3 [19,20].

Glioblastoma is currently considered the most challenging cancer to treat. The invasive nature of the disease renders surgery alone ineffective in completely removing the tumor [21]. Consequently, a comprehensive treatment approach that encompasses various modalities is being implemented. This includes maximal surgical resection, radiation therapy and concurrent and maintenance administration of temozolomide (TMZ). TMZ, along with other alkylating nitrosoureas compounds such as lomustine (CCNU) and BCNU (carmustine)-polymer wafers (Gliadel), exerts its therapeutic effect through DNA crosslinking and methylation of amino groups in DNA molecules. These processes effectively hinder transcription and replication, preventing tumor cells from proliferating and growing [22]. Additionally, the metabolite of cyclophosphamide (CPA), known as phosphoramide mustard, acts by cross-linking and alkylating DNA, thereby altering its function and impeding the growth of tumor cells [23]. Another innovative technique being employed is tumor-treating fields (TTF), which offers a novel treatment approach for both newly diagnosed and recurrent glioblastoma. Utilizing the portable medical device Optune, low-intensity, intermediate-frequency alternating electric fields are applied to disrupt the highly coordinated dividing processes in glioblastoma cells. Ultimately, this results in the rupture of cell membranes, contributing to the suppression of tumor growth [24]. Furthermore, bevacizumab, a humanized IgG1 monoclonal antibody, has been utilized in anti-angiogenic treatment strategies for recurrent glioblastomas. By binding to the VEGF ligand, bevacizumab prevents its interaction with its receptor, thus impeding angiogenesis [25]. Another humanized monoclonal antibody called nibotuzumab binds to EGFR and changes cell division patterns [26]. Additionally, depatuxizumab mafodotin (ABT-414), a monoclonal antibody-drug combination, inhibits EGFR amplification in glioblastoma and blocks tubulin polymerization through the tubulin inhibitor monomethyl auristatin F, further combating the tumor [27]. Despite these groundbreaking therapies, the clinical outcomes of advanced treatment approaches such as TTF, anti-angiogenic treatment, immunotherapy, epigenetic treatment and oncolytic viral therapy remain uncertain or even disappointing. Nevertheless, the field of medicinal chemistry has recognized the significance of heterocyclic compounds in the development of effective drugs. These compounds, characterized by their prevalent presence and versatile physicochemical properties, have emerged as indispensable components in numerous drugs available in the market [28] Several heterocyclic compounds have shown promise in the treatment of cancer, including pyrimidine, quinoline, thiazole, imidazole, indole, acridine, triazine and others. These compounds are being actively researched for their potential to treat various types of cancers, including glioblastoma [29]. Heterocyclic substances have a significant active role in the treatment of cancer, among the different therapeutic uses. In this paper, the most current advancements in heterocyclic compounds as prospective glioblastoma treatments are covered [30]. Despite comprehensive efforts, the treatment of GBMs remains a challenge for oncologists and the existing chemotherapeutic agents on the market often come with a range of side effects [31,32]. However, advancements in the treatment of GBMs have led to improvements in the quality of life and survival rates of GBM patients. Chemotherapeutic agents have been appraised to ameliorate the existing treatment of GBMs [33]. One class of chemotherapeutic agents that have shown promise in clinical studies of GBM are nitrogen-containing alkylating agents such as CCNU, BCNU and TMZ. These agents, particularly TMZ, which is an imidazotetrazine-containing heterocycle, have been found to support radiation therapy and provide benefits for patients with GBM [34].

Kinases have emerged as the primary targeted pathway in various treatment strategies. These enzymes play a crucial role in cellular processes by either removing phosphate moieties from proteins (phosphatases) or transferring phosphate groups to proteins (kinases) [35]. As a result, a significant portion of research and development efforts in the pharmaceutical industry are dedicated to designing kinase inhibitors, with approximately one-fourth of such activities focused on this area. Numerous kinase inhibitors have been developed, including well-known examples such as Imatinib, abemaciclib, afatinib, neratinib and idelalisib. Most of these inhibitors contain N-containing heterocycles [29]. For instance, afatinib, which contains a quinazoline heterocycle, has demonstrated anti-cancer efficacy in head and neck squamous cell carcinoma (HNSCC) by inhibiting the mammalian target of rapamycin (mTORC1), leading to apoptosis in cancer cells [36]. In addition to small-molecule kinase inhibitors, targeted antibodies against kinases have also shown promise in cancer treatment. Trastuzumab, for breast cancer and cetuximab, for colorectal and head and neck cancer, are examples of kinase-targeted antibodies that have exhibited efficacy in treating various types of cancers [37,38]. This shift in cancer treatment strategies, specifically targeting kinases with oncogenic, transformative and metastatic potential, has brought about significant changes in how cancer is managed. Numerous studies have demonstrated the occurrence of cytoplasmic tyrosine kinase mutations in various oncogenic situations [39]. Furthermore, primary GBM typically exhibits EGFR duplication (34%) and phosphatase and tensin homolog (PTEN) mutations (24%), in contrast to secondary GBM characterized by TP53 alterations (65%) and IDH1 mutations (70%) [40]. EGFR, particularly ErbB2, mutates in several epithelial tumors and clinical investigations showed that they contributed to the development of cancer [41,42]. Ligand binding to the receptor’s extracellular domain triggers oligomerization and often leads to receptor dimerization. This arrangement positions the tyrosine kinase domains of both receptors close to each other, thereby stabilizing the active state of the kinase. Deregulation of receptor tyrosine kinases (RTKs), resulting from genetic changes, aberrant expression and cellular distribution, has been identified as a significant contributing factor to the growth of numerous cancers, including gliomas [43].

The primary objective of medicinal chemists in the field is to devise new compounds that exhibit potential effectiveness in treating GBMs. A significant challenge lies in identifying novel structures that possess potent therapeutic properties while minimizing toxicity. In this discussion, we will provide a comprehensive summary of the molecular basis of heterocycles for GBM treatment, as well as present several emerging therapeutic pathways.

### Receptor tyrosine kinases targeting glioblastoma

In the realm of cellular signaling, phosphorylation plays a critical role, facilitated by the action of kinases, which transfer phosphate groups from ATP to specific target molecules. Among the diverse kinase enzymes, tyrosine kinases stand out as a subclass that possesses a distinct preference for phosphorylating tyrosine amino acids. With their pivotal role in signal transduction, tyrosine kinases serve as key regulators of various cellular processes, including division, growth, migration and longevity [44]. The protein structure of RTKs consists of a single transmembrane helix, providing an extracellular ligand-binding site. On the cytoplasmic side, RTKs harbor the protein tyrosine kinase domain, characterized by a tyrosine-rich carboxy-(C) terminal and the juxtamembrane regulatory region [45]. Upon activation, RTKs initiate a cascade of protein recruitment, leading to the integration of multiple signaling pathways and ultimately giving rise to distinct cellular responses. Ligand binding to an RTK stabilizes the connections between monomeric or oligomeric receptors, forming active dimers or oligomers [46,47]. Subsequently, phosphorylation of the intracellular tyrosine kinase domain creates a Src homology 2 (SH2) binding site. Such interaction between ligand and receptor triggers the phosphorylation and activation of downstream proteins, marking the initiation of well-characterized signal transduction pathways, namely the mitogen-activated protein kinase (MAPK), phosphoinositide 3-kinase (PI3K)/AKT, Janus kinase (JAK)/Signal transducers and activators of transcription (STAT) and NF-κB pathways [45]. Within the process of research and drug development, receptor tyrosine kinase inhibitors (RTKIs) represent a diverse and impactful class. Type I inhibitors, that engage in substrate competition and bind to the active conformation’s ATP-binding pocket. Cabozantinib, crizotinib, gefitinib and vandetanib are a few examples [48]. On the other hand, Type II inhibitors target the inactive state of protein kinases and typically lack selectivity. Prominent representatives encompass imatinib, axitinib and sorafenib [49]. Allosteric inhibitors, classified as Type III inhibitors, bind near the ATP-binding pocket and exhibit a high degree of selectivity. Notable among this category are GnF2 and trametinib [50]. Type IV inhibitors, referred to as substrate-directed inhibitors, demonstrate specificity towards kinases and engage in reversible interactions outside of the ATP pocket. ONO12380 serves as an illustrative example [51]. Finally, Type V inhibitors form an irreversible covalent bond with their protein kinase target, resulting in a potent and irreversible binding. Prominent representatives include HK1-272, ibrutinib and afatinib [52].

There is significant research being conducted on various RTKs for the treatment of cancer. Among these, the epidermal growth factor receptor (EGFR) stands out as it is highly expressed in GBM [41]. The main ligands for EGFR, a transmembrane tyrosine kinase with a weight of 170 kDa, are epidermal growth factor (EGF) and transforming growth factor (TGF-α) [53]. The HER1 (human EGFR-related) or ErbB1 (erythroblastic oncogene B) transmembrane receptor of tyrosine kinase, along with HER2/neu (ErbB2), HER3 (ErbB3) and HER4 (ErbB4), are members of the ErbB family of RTKs and are crucial for both healthy physiology and malignancies [54,55]. In the conventional subtype of glioma, EGFR amplification is observed. Around 57.4% of individuals with primary GBM exhibit EGFR gene amplification, resulting in elevated levels of EGFR protein and promoting tumor proliferation and growth [41]. One noteworthy EGFR mutation, known as EGFR variant III (EGFRvIII), has been observed in 30% to 50% of gliomas with increased EGFR expression. Experimental evidence suggests that EGFRvIII enhances mitogenic effects, activates pro-invasive and anti-apoptotic signaling pathways and contributes to tumor progression [56]. The family of RTKs also includes the vascular endothelial growth factor receptor (VEGFR) that has three subtypes, VEGFR1, VEGFR2 and VEGFR3, there are five structurally similar VEGF ligands, including VEGFA, VEGFB, VEGFC, VEGFD and placenta growth factor (PIGF) [57]. Normal VEGFR activation leads to biological activities such as angiogenesis, endothelial cell migration, lymphangiogenesis and fatty acid absorption. Differential expression of VEGFR1, VEGFR2 and VEGFR3 is observed in various stages of cervical, prostate and ovarian cancer, with VEGFR3 often being overexpressed in advanced stages [58]. The FGFR1, FGFR2, FGFR3 and FGFR4 genes together encode seven distinct forms of fibroblast growth factor receptors (FGFRs). FGFR signaling is crucial for both adult development and embryonic development [59]. Cancer-associated alterations in FGFRs are widespread, with FGFR1 being the most frequently mutated gene (49%), followed by FGFR3 (23%), FGFR2 (19%) and FGFR4 (7%). Amplification of FGFR1 in lung and breast cancer cells has been shown to enhance MAPK and PI3K signaling pathway activation, ligand-dependent signaling and the expression of stem cell markers [60]. Additionally, oncogenic gene fusions involving FGFR2 and FGFR3 are frequently observed. In glioblastoma, the fusion of FGFR3 with transforming acidic coiled-coil containing protein (TACC3) results in FGFR3-TACC3, which activates MAPK and extracellular signal-regulated kinase (ERK) signaling, facilitates *in vitro* transformation and increases cell proliferation [61]. The platelet-derived growth factor receptor (PDGFR) is a crucial subfamily of RTKs, comprising PDGFRα and PDGFRβ, also known as PDGFRA and PDGFRB, respectively. Activation of these receptors occurs through binding with five different PDGF ligands, namely PDGF-AA, PDGF-AB, PDGF-BB, PDGF-CC and PDGF-DD [62]. Exciting recent findings have revealed the overexpression of the PDGFRA gene in oral squamous cell carcinoma, which has been associated with metastasis and reduced patient survival [63]. Additionally, PDGFR overexpression has been implicated in the co-amplification of other RTKs and could serve as a prognostic marker in medulloblastomas, lung cancer and ovarian cancer. In these malignancies, the co-expression of PDGFR and its ligands drives an autocrine loop that activates downstream pathways such as PI3K/AKT, MAPK and STAT [64]. Moving on, the insulin receptor (IR) subfamily of RTKs consists of IGF-1R, IRA, IRB, IGF-1R/IR (hybrid) and IGF-2R. While extensive research has been conducted on IGF-1R and IGF-2R, it is worthy to note that IGF-1 and IGF-2 act as ligands to activate IGF-1R. In contrast, IGF-2R functions as a non-signaling receptor responsible for facilitating the removal of IGF-2 from the cell surface [65]. Elevated expression of IGF-1R has been observed in various cancers, including breast, prostate, lung, pancreatic and colon cancers [66]. Differing patterns of IGF-1R expression have been associated with distinct clinical implications. Higher levels of cytoplasmic IGF-1R have been linked to an increased risk of recurrence following radiotherapy, whereas elevated overall IGF-1R levels correspond to higher tumor grades. The overexpression of IGF-1R during tumor development activates downstream signaling pathways such as AKT, ERK1/ERK2 and STAT3 [67]. Furthermore, we turn our attention to the MET gene, which encodes the hepatocyte growth factor receptor (HGFR), commonly known as MET or c-Met. Its ligand, hepatocyte growth factor (HGF), is vital in promoting cellular responses. Cancers of the breast, lung, ovary, colon, cervical, kidney and blood have demonstrated overexpression of c-Met [68]. Breast cancer cases featuring c-Met overexpression often present with high histologic grades, distant metastases and large tumor sizes. Interestingly, hepatocellular carcinoma has exhibited resistance to multi-kinase inhibitors through autocrine stimulation of HGF/c-Met signaling. Studies have discovered that resistant cells exhibit elevated HGF levels and activated c-Met and blocking this signaling pathway reduces the migratory and invasive abilities of these cells [69].

Tyrosine kinase receptors share a common structural framework whereby ligand binding to receptors such as EGFR, VEGFR, PDGFR and FGFR leads to their translocation to the nucleus. Once in the nucleus, these receptors exert their influence on gene transcription by interacting with two significant downstream signaling pathways: RAS/MAPK/ERK and RAS/PI3K/AKT. These pathways play crucial roles in various cellular processes including proliferation, invasiveness, survival and angiogenesis [70]. Specifically, the activation of FGFR4 facilitates the regulation of the epithelial-to-mesenchymal transition (EMT). This activation not only impacts the expression of E-cadherin, a vital adhesion protein for epithelial cells, but also leads to the phosphorylation of adaptor proteins linked to the RAS/RAF/MEK/ERK and PI3K/AKT signaling pathways. Consequently, FGFR4 activation plays a critical role in cell plasticity and cell motility [71]. IGF-2R is the monomeric insulin receptor, is not engaged in signal transduction and does not have tyrosine kinase activity. Instead, IGF-2R contributes to the trafficking of lysosomal enzymes towards lysosomes [72]. Furthermore, even in the presence of EGFR antagonists in combination with c-Met inhibitors, ErbB3 interaction and reactivation of the PI3K/AKT pathway may cause c-Met to give rise to acquired resistance to small-molecule EGFR inhibitors like gefitinib [73]. This finding highlights the complexity of targeted therapies and the need for further research to overcome acquired resistance mechanisms. Overall, the dysregulation of signaling pathways plays a crucial role in the development and progression of various cancers. Understanding the interplay between these receptors, their ligands and downstream signaling pathways could pave the way for the development of targeted therapies to combat these malignancies effectively. Continued research into the molecular mechanisms underlying these aberrant signaling pathways will undoubtedly contribute to advancements in cancer treatment and patient care.

### Receptor tyrosine kinase signaling pathways

**Figure 2:**
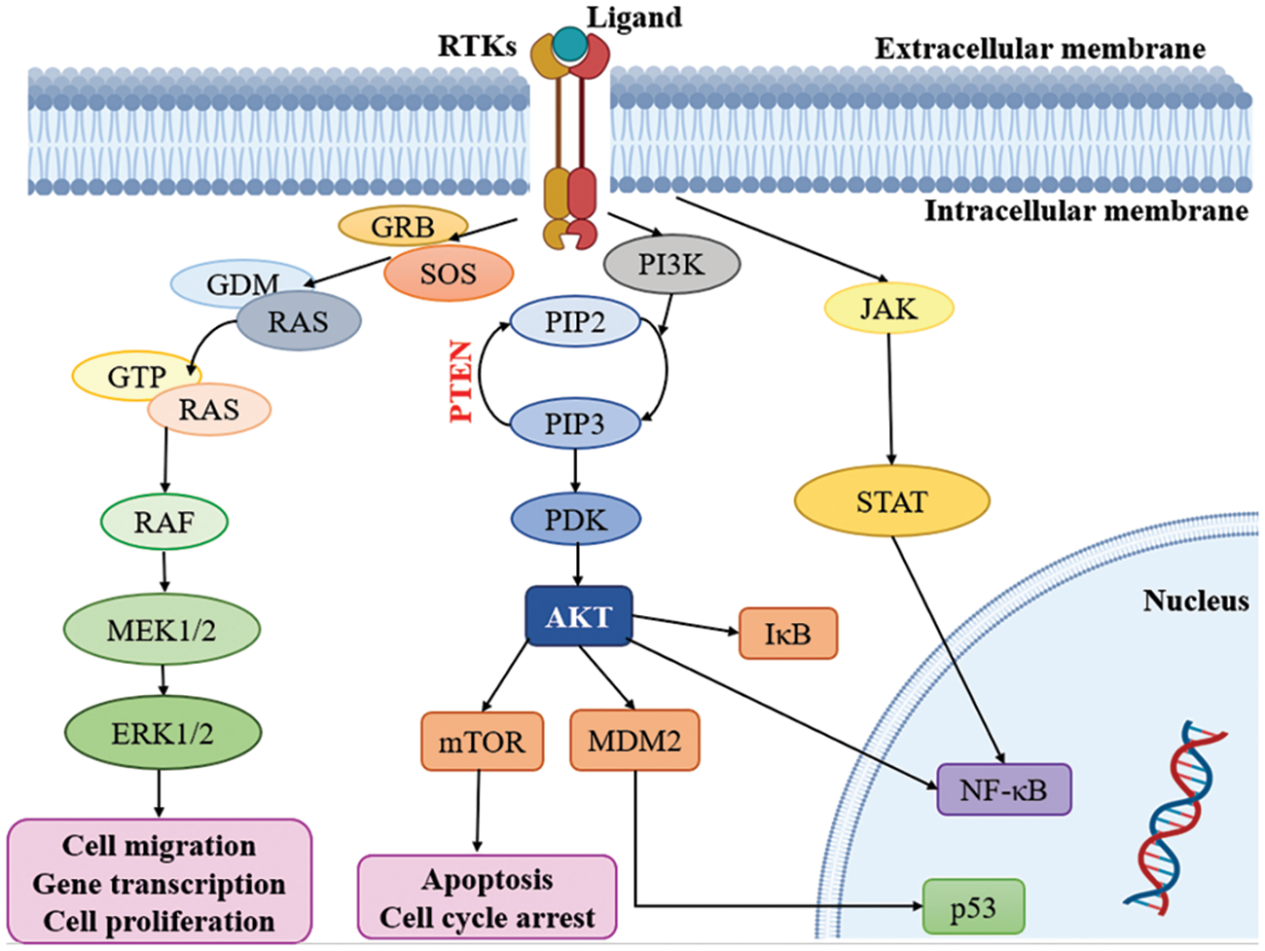
Kinases targeting pathways in glioblastoma. RTKs: Receptor tyrosine kinases; GRB: Growth factor receptor binder; SOS: Son of sevenless; MEK: Mitogen-activated protein kinase; ERK: Extracellular-signal-regulated kinase; PI3K: Phosphatidylinositol 3-kinase; PIP2: Phosphatidylinositol 4, 5-bisphosphate; PIP3: Phosphatidylinositol 3, 4, 5 trisphosphate; PDK: Phosphoinositide-dependent protein kinase; mTOR: mammalian target of rapamycin; MDM2: Mouse double minute 2; JAK: Janus kinase; STAT: Signal transducers and activators of transcription.

Multiple signaling cascades are activated because of the activation of RTKs, leading to various findings. Modify the expression of RTKs, their ligands and related proteins, focused on the MAPK, PI3K/AKT, JAK/STAT and NF-κB downstream pathways. Subsequently, all the pathways, including RAS-RAF-MAPK, PI3K and AKT, are activated [53,74]. Notably, the RAS and PI3K signaling pathways are recognized as key contributors to the development of malignancies within oncogenic pathways, surpassing other pathways in their significance (Fig. 2) [75]. It is important to highlight the role of the PI3K/mTOR signaling pathway in regulating fundamental biological processes such as cell metabolism, growth, survival and motility [76]. Moreover, the RAS/MAPK/ERK and RAS/PI3K/AKT pathways serve as vital downstream pathways for RTKs, promoting uncontrolled cell proliferation, angiogenesis, metastasis and chemoresistance [77,78]. RTK signaling at the plasma membrane involves the activation of multiple pathways, including PI3K/protein kinase B (PKB/AKT), JAK/STAT and protein kinase C (PKC), in addition to the RAS/MAPK/ERK pathway [79,80]. Given the complexity of these signaling pathways, it is crucial to further explore their roles in glioblastoma. In-depth analysis of their involvement will provide valuable insights into the mechanisms underlying this aggressive form of brain cancer.

The external membrane ligand initiates cellular signaling on the cell surface through the activation of RTKs. This activation leads to the recruitment of guanine exchange factors, which promote the loading of RAS proteins with GTP. Subsequently, RAF proteins or RAF/MEK heterodimers are recruited to the plasma membranes by RAS-GTP dimers or nanoclusters [81]. Once at the plasma membrane, RAF and MEK proteins form temporary tetramers that facilitate RAF activation through back-to-back dimerization. MEK proteins, in turn, dock on activated RAF dimers, forming face-to-face homodimers that are activated by RAF. This activation results in the phosphorylation of ERKs, which elicits a downstream cellular response [82]. In addition to the RTK-mediated pathway described above, the PI3K/AKT pathway also plays a crucial role in the regulation of cell invasion, migration and apoptosis. Phosphorylation of RTKs leads to the activation of PI3K, initiating a signaling cascade [74]. Notably, the tumor suppressor gene PTEN (Phosphatase and Tensin homolog) is frequently mutated or genetically altered in glioblastomas, leading to dysregulation of this signaling pathway. PTEN serves as an important negative regulator of the PI3K/AKT pathway. Mutational inactivation of PTEN has been observed in 20% to 40% of glioblastomas, while promoter methylation results in genetic loss in approximately 35% of cases [83]. Moreover, the JAK/STAT pathway acts as a link between extracellular cues and nuclear transcriptional responses. Although primarily associated with the activation of non-receptor tyrosine kinases and RTKs, STAT proteins can also be phosphorylated by these signaling molecules. Notably, several signaling pathways, including PI3K/AKT, JAK/STAT and RAS/MAPK, have been implicated in the activation of NF-κB, which is frequently observed in glioblastomas [80].

### Mitogen-activated protein kinase

The MAPK pathway is a crucial mechanism that governs various cellular processes, including cellular proliferation, differentiation, growth, apoptosis and transformation. This pathway comprises several families of MAPKs, namely ERK, JNK and p38, which have been extensively studied. Various stimuli such as growth factors, cytokines, stress and ceramides can activate the MAPK pathway [84]. In the typical cascade of MAPK signaling, a series of activation events occur sequentially. First, a MAPK kinase kinase (MAPKKK) is activated, followed by the activation of a MAPK kinase (MAPKK) and ultimately resulting in the activation of the MAPK itself [45]. It is worth noting that patients with glioblastomas frequently exhibit activated RAS pathways and elevated levels of RAS-GTP [56]. One well-known MAPK signaling pathway is the Raf-Mitogen-activated protein kinase (MEK)-ERK pathway. In this pathway, a growth factor or ligand binds to the cell surface RTK, leading to RTK dimerization and subsequent transphosphorylation [85]. Subsequently, a protein containing the Src homology domain, such as growth factor receptor binder-2 (GRB2) with its SH2 domain, interacts with the RAS activator son of sevenless (SOS). This interaction ultimately leads to the activation of c-RAF (a MAPKKK), MEK1/2 (a MAPKK) and finally ERK1/2 (a MAPK) [86] [87]. Stress-related factors, such as DNA damage, UV radiation and inflammation, primarily activate the JNK proteins, also known as stress-activated protein kinases (SAPKs). The activation of JNK involves direct phosphorylation by MAPKKs, specifically MKK4 and MKK7, as well as additional phosphorylation by MAPKKKs. Furthermore, the p38 kinase consists of four isoforms (α, β, γ and δ) and their activation is mediated by MAPKKs, which are in turn phosphorylated by MAPKKKs [84,88]. In summary, growth factors predominantly activate the ERK1/2 cascade, while JNK activation is relatively weaker and p38 activation occurs less frequently. The MAPK pathway plays a crucial role in regulating various cellular processes and its dysregulation has been implicated in numerous pathological conditions.

### Phosphoinositide 3-kinase/AKT pathway

The PI3K-AKT pathway is a significant signaling system that plays a crucial role in maintaining cellular homeostasis, metabolism, growth and macromolecule production. It is primarily activated by growth factors, insulin and cytokines [89]. Although there are three different classes of PI3K enzymes, only class I PI3Ks are relevant to cancer. The four distinct isoforms of Class I PI3Ks (p110α, β, γ and δ) are recognized as PIK3CA, PIK3CB, PIK3CG and PIK3CD [90]. Alterations in the EGFR and PI3K/AKT/rapamycin-sensitive mTOR pathway have been frequently observed in the development of GBM [91]. Under normal physiological conditions, PI3K activation at the plasma membrane results in the phosphorylation of phosphatidylinositol 4, 5-bisphosphate (PIP2) to generate phosphatidylinositol 3, 4, 5 trisphosphate (PIP3), which acts as a second messenger [92]. Complex 1 (mTORC1) and Complex 2 (mTORC2) are two distinct mTOR complexes with distinct localizations and roles. PI3K-dependent or PI3K-independent pathways can directly activate mTORC1, which comprises the scaffolding protein RAPTOR (regulatory-associated protein of mTOR), which controls cell growth [76]. On the other hand, mTORC2 phosphorylates AKT at Ser-473, contributing to cell longevity, metabolism, growth and cytoskeletal structure [40,93]. The activity of the AKT isoforms (AKT1, AKT2 and AKT3) is enhanced through phosphorylation by mTOR complex 2 (mTORC2) and phosphoinositide-dependent protein kinase 1 (PDPK1/PDK1) [90]. The dephosphorylation of the secondary messenger PIP3 to PIP2 allows PTEN to block the PI3K pathway. Acting as a lipid phosphatase, the tumor suppressor PTEN converts PIP3 into PIP2, preventing the activation of AKT [40]. However, in the case of GBM, continuous activation of the PI3K/AKT/mTOR signaling pathway occurs due to mutations in the EGFR or PTEN, leading to carcinogenesis and resistance to cancer therapy [74]. Furthermore, the cell cycle and proliferation are regulated by tumor suppressor genes such as TP53, p16 and PTEN phosphatase. The alteration or loss of these cancer suppressor genes contributes to the initiation and formation of gliomas [83] TP53, which is the most frequently dysfunctioning gene in cancer, is dysregulated in 94% of GBM cell lines and 84% of glioblastoma patients. Downregulation of components in the p53 pathway has been associated with GBM cell invasion, migration, proliferation, evasion of caspase-mediated apoptosis and maintenance of cancer stemness [94]. p53 plays a crucial role in tumor suppression by controlling transcription and engaging in nuclear-cytoplasmic shuttling, thus promoting DNA repair and inducing apoptosis [95]. In addition to its role in cellular signaling, mTOR also plays a significant role in regulating autophagy. Negative regulators such as AMPK and p53 restrict mTOR activity, promoting the progression of the autophagy process. On the other hand, AKT and MAPK signaling activate mTOR, suppressing autophagy signaling [96].

### JAK/STAT pathway

One of the key mechanisms involved in RTK signal transduction is the JAK-STAT pathway. This pathway is triggered by a range of cytokines, interferons and other associated components [97]. The connection between four JAKs—JAK1, JAK2, JAK3 and TYK2—and seven STATs—STAT1, STAT2, STAT3, STAT4, STAT5a, STAT5b and STAT6—allows direct transmission from the membrane to the nucleus. Upon ligand binding to a receptor, receptor-associated JAKs are activated and initiate cross-phosphorylation of each other and the intracellular region of their respective receptors [98]. This process creates docking sites for the recruitment of cytoplasmic STATs. Once activated by JAK phosphorylation, STATs translocate to the nucleus where they bind to DNA and regulate gene expression [99]. Numerous RTKs connect with the JAK/STAT pathway to promote cell proliferation and differentiation. However, it is noteworthy that JAK mutations are frequently observed in leukemia and other solid tumors, facilitating the invasion, proliferation and migration of cancer cells [100]. Similarly, STAT3 and STAT5 mutations are also commonly found in a variety of malignancies. Paradoxically, research has demonstrated that despite their contradictory nature, both STAT3 and STAT5 can function as tumor suppressors [101]. STAT3 acts as a transcription factor, promoting the production of genes involved in signal transduction, angiogenesis, cell proliferation and anti-apoptotic responses. GBMs exhibit high levels and sustained activation of STAT3, suggesting its key role in regulating GBM aggressiveness. Furthermore, abnormal activation of tyrosine kinases JAK1 and JAK2, which are crucial for STAT3 activation, has been observed in GBMs [102]. In addition, the overexpression of protein kinase CK2, which potentiates JAK1, JAK2 and STAT3 activation, is seen in GBMs. This correlates with the absence or extremely low expression of Protein Inhibitor of Activated STAT3 (PIAS3), a negative regulator of activated STAT3, in GBMs [103]. In GBM, STAT5 signaling has been connected to tumor development. Granulocyte-macrophage colony-stimulating factor (GM-CSF), a substance released by glioma cells, stimulates STAT5 signaling in myeloid-derived suppressor cells (MDSCs), causing Bcl-2 expression and downregulation of IRF8 transcription, ultimately preventing apoptosis and promoting proliferation [104].

### Nuclear factor kappa B

The transcription factors (TFs) that comprise nuclear factor kappa B (NF-κB) govern various biological processes, such as inflammation, cell division and apoptosis [105]. The five members of the NF-κB family are p50/NF-κB1, p52/NF-κB2, Rel-like domain-containing protein A (RelA/RELA), RelB/RELB and c-Rel/REL. NF-κB is a dimeric DNA-binding complex with different combinations of family members [106]. Unregulated NF-κB signaling in gliomas has been associated with many pathways. For instance, several signaling pathways, such as RAS/MAPK, PI3K/AKT and JAK/STAT activation, have been linked to the activation of NF-κB, mainly the EGFR and PDGFR, which are frequently present in GBM [107]. Tumor suppressors including PTEN and neurofibromin 1 (NF1) have been linked to abnormal activation of NF-κB in GBM. Through the development of proteins that block the death receptor apoptotic pathway, inhibitors of apoptosis (IAPs), anti-apoptotic Bcl-2 family members and other anti-apoptotic proteins, NF-κB activation in cancer can result in apoptosis resistance [108]. As a result of PKC and mTOR signaling, NF-κB activity also speeds up the cell cycle by causing the production of important cell cycle proteins like cyclin D1 in GBM cells [109]. Deregulated RTK/NF-κB signaling has been connected to several cancers [110].

### Heterocycles targeting protein kinases

Researchers are creating a wide variety of heterocycles and testing them for anticancer properties. A few of them can be investigated further for clinical trials as they exhibit good inhibitory action. Some heterocycles that target tyrosine kinase downstream pathways and are effective against glioblastoma have been discussed below.

### Pyrimidine-containing heterocycles

Over the past ten years, TKIs have been approved for the treatment of many malignancies (imatinib, gefitinib, erlotinib, sunitinib and sorafenib). These compounds usually possess a scaffold that is either pyrimidine-or pyrrolo-pyrimidine-based, or one that is substantially similar to one. Pédeboscq et al. [111] designed ten thienopyrimidine compounds with a structure that is remarkably close to that of commercially available medications. The cytotoxic effect of the compounds was evaluated using the flow cytometry method and the MTT assay on glioblastoma cell lines. Two molecules displayed significant cytotoxicity towards EGFR when compared with gefitinib (IC_50_: gefitinib = 51.9, **1 =** 61.8 ± 0.9 and **2 =** 41.2 ± 1.2 μM), indicating blockage of the EGFR pathway via interacting with the TK receptor. Abdelhaleem et al. [112] reported the synthesis of many new tetrahydrobenzothieno [2,3-d] pyrimidine urea derivatives using a fragment-based design technique. The MCF-7 cell line was used to test their anticancer efficacy. This study discovered the small-molecule inhibitor **3,** which had the strongest anti-proliferative effects. This molecule’s anti-proliferative action appears to be directly linked to its ability to inhibit topoisomerase II (IC_50_ = 9.29 μM). Furthermore, compound **3** showed good suppression of VEGFR-2, with an IC_50_ value of 0.2 μM, 2.1 times greater than that of sorafenib. Moreover, activation of the deoxyribonucleic acid (DNA) damage response system leads to cell cycle arrest at the G2/M phase and cell accumulation in the pre-G1 phase after staining of cells with annexin-V and propidium iodide (PI). This mechanism was supported by a notable increase in the Bax/Bcl-2 ratio, a significant rise in the quantity of active caspase-3 and a substantial improvement in the expression of the tumor suppressor gene p53. In further studies, a member of the Ser/Thr kinase family, Fas-Activated Serine/Threonine Kinase (FASTK) has been linked to cancer development and apoptotic evasion. In light of this, a range of novel thienopyrimidine-based chalcones were synthesized by Khan et al. [113] and their capacity to affect FASTK-mediated apoptotic evasion was examined. Out of 15 synthesized compounds, initial screening using enzyme inhibitory assays and binding studies for FASTK revealed three thienopyrimidine-based chalcone analogs having significant binding affinities and enzyme inhibitory ability in the nM range. HEK-293 and MCF-7 cells were used to evaluate the effects of these three molecules on cell growth. For MCF-7 cells, the IC_50_ values of compounds **4**, **5** and **6** were reported to be 20.22 ± 1.50, 6.52 ± 0.82 and 8.20 ± 0.61 µM, respectively. According to annexin-V and PI labeling, the aforementioned compounds stop the cell cycle at the G0/G1 phase, induce programmed cell death in MCF-7 cells and subsequently impede cell migration by blocking the production of FASTK and reactive oxygen species.

In patients with glioblastomas, overexpression and activation of focal adhesion kinase (FAK) promote tumor growth and invasion. Thus, FAK kinase could represent a novel therapeutic approach for glioblastoma. Li et al. [114] made several brand-new 2,4-diaminopyrimidines and 2-amino-4-arylmethylaminopyrimidines covalent inhibitors of the FAK kinase in this work. To find out how selective these covalent inhibitors were, the compounds were put to the test against a variety of kinases. These new compounds all have IC_50_ values in the nanomolar range, with compound **7** showing a very potent inhibitory effect against the FAK enzyme at 5.5 ± 0.4 nM. Only two kinases, FAK and Pyk2, were significantly inhibited by compound **7**, which also had a favorable selectivity profile. They also significantly reduced the rate of cell migration and the progression of the cell cycle in U87 cells by stopping them from continuing through it. Furthermore, this inhibitor significantly and dose-dependently decreased the autophosphorylation of NF-κB, FAK and its downstream effectors, AKT and ERK, in glioblastoma cells. As new FAK inhibitors, Wang et al. [115] developed and invented an array of 2,7-disubstituted-thieno [3,2-d] pyrimidine analogs. Most of the compounds were able to successfully inhibit the proliferation of MDA-MB-231, A-549 and U-87MG cancer cell lines as well as the enzymatic activity of FAK. Out of these analogs, compound **8** demonstrated significant activity as compared to TAE-226 in A-549, U-87MG and MDA-MB-231 cells, with IC_50_ values of 0.27, 0.16 and 0.19 µM, respectively. Compound **8** (IC_50_ = 3.32 µM) exhibited lower toxicity towards the HK2 human cell line. The flow cytometry results showed that compound **8** successfully halted MDA-MB-231 cells in the G0/G1 phase and it also dose-dependently increased apoptosis in the same cells.

Using human glioblastoma cells, Koul et al. [116] investigated the preclinical effectiveness and clinical development potential of several drugs in both *in vitro* and *in vivo* approaches. Compound **9** was found to be a specific pan-class I PI3K inhibitor. A 72-h treatment with compound **9** caused dose-dependent growth suppression and successfully shut down the PI3K/AKT signaling cascade. Compound **9** showed different forms of cell death depending on the p53 status of the cells: p53 mutant cells died through mitotic damage, while p53 wild-type cells died by apoptosis. Additionally, Ibrahim et al. [117] synthesized four series of condensed pyrrolo [1,2-c] pyrimidines as PI3K inhibitors. PI3K kinase inhibitory activity was observed at low micromolar or nanomolar concentrations of the examined compounds. The morpholino-pyrimidopyrrolopyrimidinones series and the morpholino-pyridopyrrolopyrimidine-2-carbonitriles series were found to be extremely strong and selective PI3K inhibitors (IC_50_ = 0.1–7.7 nM). The target substances significantly reduced the viability of the p110α-overexpressed HeLa cervical cancer cell line (IC_50_ = 0.21–1.99 μM). A simulation of molecular modelling demonstrated that the suggested chemicals effectively docked with the p110 active site. Multiple beneficial H-bond and hydrophobic interactions within the enzyme-binding pocket stabilized the complexes. However, Zhu et al. [118] developed several 7,8-dihydro-5H-thiopyrano [4,3-d] pyrimidine derivatives. The inhibitory potential of every drug was evaluated at a concentration of 10 μM. A few selected compounds were subjected to additional testing to ascertain their IC_50_ values for two cancer cell lines and the mTOR kinase, as well as their inhibitory action against PI3Kα at a 10 μM level. The most promising molecule **10** demonstrated substantial anticancer effects against H460, mTOR kinase and PC-3 cell lines with IC_50_ values of 7.43 ± 1.45, 0.80 ± 0.15 and 11.90 ± 0.94 μM, respectively. Compared to the lead compound BMCL-200908069-1 (9.52 ± 0.29, 1.37 ± 0.07 and 16.27 ± 0.54 μM), it was 1.28 to 1.71-fold more active, respectively. The docking was performed with mTOR as a docking model (PDB ID: 4JT6). A novel panel of 7,8-dihydro-5H-thiopyrano [4,3-d] pyrimidine analogs with a chromone functional group were designed and synthesized by Sun et al. [119]. To test the IC_50_ values of all the molecules five cancer cell lines were used (A-549, PC-3, MCF-7, HeLa and HepG-2) and displayed activity with IC_50_ in a range of 34.9–0.17 μM. The inhibitory effects of these drugs on mTOR kinase were assessed. Furthermore, four of them also underwent additional evaluations for their effects on PI3Kα kinase. The outcomes showed that compound **11** exhibited outstanding inhibitory and cytotoxic action against mTOR kinase and PI3Kα kinase with IC_50_ values of 1.1 ± 0.10 and 0.92 ± 0.12 μM, respectively.

Recently, pyrrolo-pyrimidine analogs have been approved by the FDA in the US and other countries for the treatment of a variety of illnesses after undergoing phase I/II clinical research. Musumeci et al. [120] described the synthesis, characterization and anti-GBM activity of numerous novel pyrrolo [2,3-d] pyrimidine compounds. These compounds interacted with the ATP binding site of Src family kinases (SFKs) in an efficient manner, as demonstrated by docking studies and MM-GBSA analysis. Enzyme tests against certain kinases revealed pyrrolo [2,3-d] pyrimidines to have a very high selectivity for Src. The U87 GBM cell line was employed for the evaluation of the antiproliferative efficacy of these compounds. Compound **12** displayed a high level of cytotoxicity with an IC_50_ value of 7.1 μM. Targeting SFKs may be a potential treatment for the most common soft-tissue sarcoma in children known as rhabdomyosarcoma (RMS). Casini et al. [121] evaluated the recently synthesized selective SFK inhibitors of pyrazolo [3,4-d] pyrimidine derivatives on RMS cell lines. Compound **13** exhibited no effect on normal cells such as primary human skin fibroblasts and differentiated C2C12 cells, however it significantly decreased cell viability and accelerated apoptosis in SFK. This suggests that compound **13** is most effective against this particular SFK member. Compound **13** also slowed tumor growth and inhibited cell migration and invasion in an RMS xenograft model. SFK inhibition influenced the NOTCH3 receptor-p38 MAPK axis, which controls the equilibrium between growth and division. In addition to this, it also led to muscle differentiation in RMS cells. Overall, the findings suggested that, in addition to reducing RMS cell proliferation and invasive potential, SFK inhibition may be used as a differentiation therapy strategy. Compound **13** was found to have an IC_50_ of 24.2 μM for C2C12 cells.

**Figure 3:**
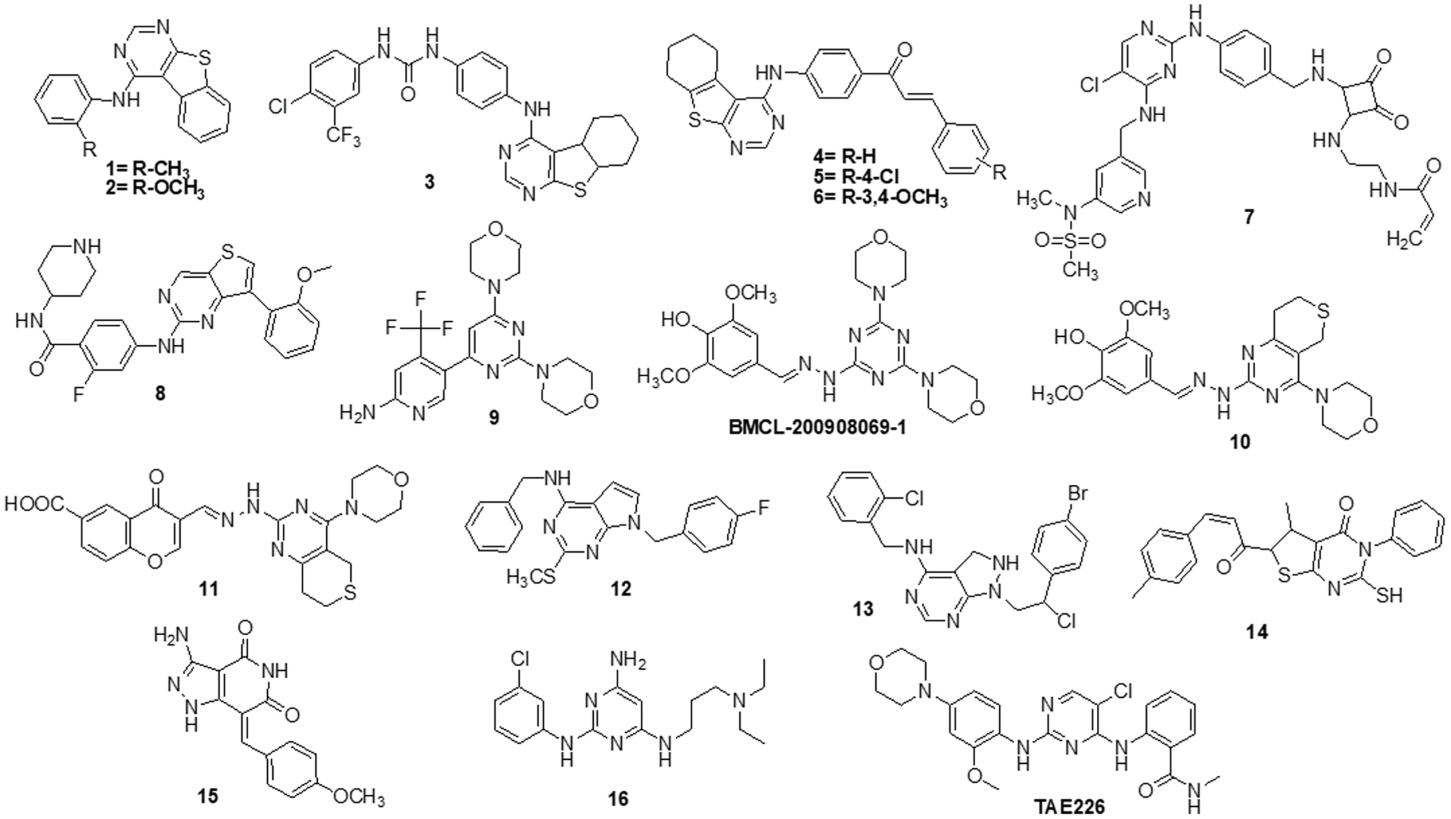
Chemical structures of pyrimidine containing heterocycles.

Fouad et al. [122] used synthetic methods to synthesise novel thiophene and thienopyrimidine compounds. The following five cell lines were used to evaluate twenty-three different substances: HepG-2, Hep-2, MCF-7, PC-3 and HeLa. The results demonstrated that some different chemicals exhibited the most anticancer efficacy against all tested cell lines in comparison to doxorubicin (DOX). Further analysis of the data showed that compound **14** was more effective than standard DOX, with IC_50_s of 7.71, 5.40 and 4.30 µM in the PC-3, Hep-2 and HeLa cell lines, respectively. Also, compound **14** was found to be the strongest tyrosine kinase inhibitor with an IC_50_ of 135.5 μM. Metwally et al. [123] reported on novel pyrazolo [4,3-c] pyridine derivatives and assessed them against several cancer cell lines, such as MCF-7, HepG-2 and HCT-116. Cytotoxic activity data showed that compound **15** is highly active towards MCF-7 and HepG-2 cells with IC_50_ values of 1.937 and 3.695 μg/ml, respectively, when compared to DOX with IC_50_ values of 2.527 and 4.749 μg/ml. New derivatives with a nitrogen-containing scaffold were invented and synthesized by Taglieri et al. [124]. Further, these compounds were tested for antiproliferative effects in tumor cells. Three novel entities were examined using the cell lines MDA-MB-231,HT-29 and U-87MG. Compound **16** significantly inhibited cell line proliferation, whereas the other chemicals had no effect on it. Furthermore, compound **16** inhibited the G0/G1 phase of the cell cycle and elevated p21 and p27 at 20 µM. When utilized at higher doses (30–50 µM), it also triggered apoptosis in all three cell lines (Fig. 3).

### Quinoline, isoquinoline, quinoxaline and quinazoline containing heterocycles

Zhang et al. [78] designed nine compounds with a benzimidazoisoquinoline moiety and tested their anticancer effectiveness on the U-87 and LN-229 glioblastoma cell lines. Compound **17** demonstrated potent anticancer activity against U-87 and LN-229, with IC_50_ values of 24.9 and 27.1 μM, respectively. Moreover, the mechanism underlying the mechanistic cell cycle arrest of glioblastoma cells at the S phase was revealed by means of flow cytometry. A PI/Annexin V-fluorescein isothiocyanate (FITC) assay results reported to caused apoptosis in U-87 and LN-229 cells in a dose-dependent way. Additionally, it was reported that the molecule has inhibitory effects on the AKT and ERK signaling pathways in glioblastoma cells. Furthermore, the growth-related markers phosphorylated (p)-AKT and p-ERK were substantially reduced in U-87 and LN-229 cells. Suppression of PI3K/AKT and RAF/MEK/ERK pathways leads to the inhibition of AKT phosphorylation and exhibits antiproliferative action in glioblastoma cells. A novel and straightforward process for making fused tricyclic pyrazoles with highly substituted isoquinoline-5,8-diones was brought out by Bertuzzi et al. [125]. Two of these scaffolds were examined by molecular docking experiments *in silico* as prospective anti-cancer medications. These scaffolds were developed to interact with a variety of biological residues. U-251, DBTRG and U-87MG cell lines were used to study the cytotoxic effects of the compound. Compound **18** was the most active of all of them, showing an IC_50_ value of around 2.5 μM after 72 h. Compound **18** was found to be capable of inhibiting PI3K/mTOR kinases, which oversee controlling a variety of cellular functions in human cancer cells. In this study, Jin et al. [126] designed and produced several new quinoline analogs of ursolic acid (UA) that containing oxadiazole, hydrazide, or thiadiazole moieties. The anticancer activities of these compounds against MDA-MB-231, HeLa and SMMC-7721 cell lines were then assessed *in vitro*. A few compounds demonstrated significant efficacy against a minimum of one cell line. Compound **19** had the strongest activity with IC_50_ values of 0.12 ± 0.01, 0.34 ± 0.03 and 0.08 ± 0.01 μM for MDA-MB-231, SMMC-7721 and HeLa. Compound **19** stopped the cell cycle at the G0/G1 phase in HeLa cells, demonstrating apoptotic action. Additionally, it strongly suppressed MEK1 kinase activity and the RAS/RAF/MEK/ERK pathway. Western blot analysis was performed to evaluate the protein expression of phosphorylated ERK, which revealed that levels of phosphorylated ERK dropped by up to 74.7%, 41.3% and 29.5% at concentrations of 0.05, 0.1 and 0.2 μM, respectively. A molecular docking investigation against the MEK receptor was conducted (PDB: 3EQF). In further studies, Aly et al. [127] developed a new family of fused naphthofuro [3,2-c] quinoline-6,7,12-triones and pyrano [3,2-c] quinoline-6,7,8,13-tetraones that target ERK inhibitors. Their ability to inhibit ERK1/2 was assessed using an *in vitro* radioactive kinase assay. ERK1 and ERK2 were both suppressed by derivatives **20** and **21,** with IC_50_ values of 0.5 and 0.19 µM and 0.6 and 0.16 µM, respectively. Kinetic mechanism tests showed that compound **21** inhibited ERK2 with a Ki of 0.09 µM, proving that they are ATP-competitive inhibitors. Additionally, they also inhibited the proliferation of BRAF mutant A-375 melanoma cells with IC_50_ values of 3.7 and 0.13 µM. The A-375 cells when treated with **20** and **21** they reduced the phosphorylation of ERK substrates p-90RSK and ELK-1 in a dose-dependent manner promoting apoptosis. Studies carried out using molecular docking technology verified the pattern of compound binding to the ERK kinase catalytic site (PDB: 4O6E).

**Figure 4:**
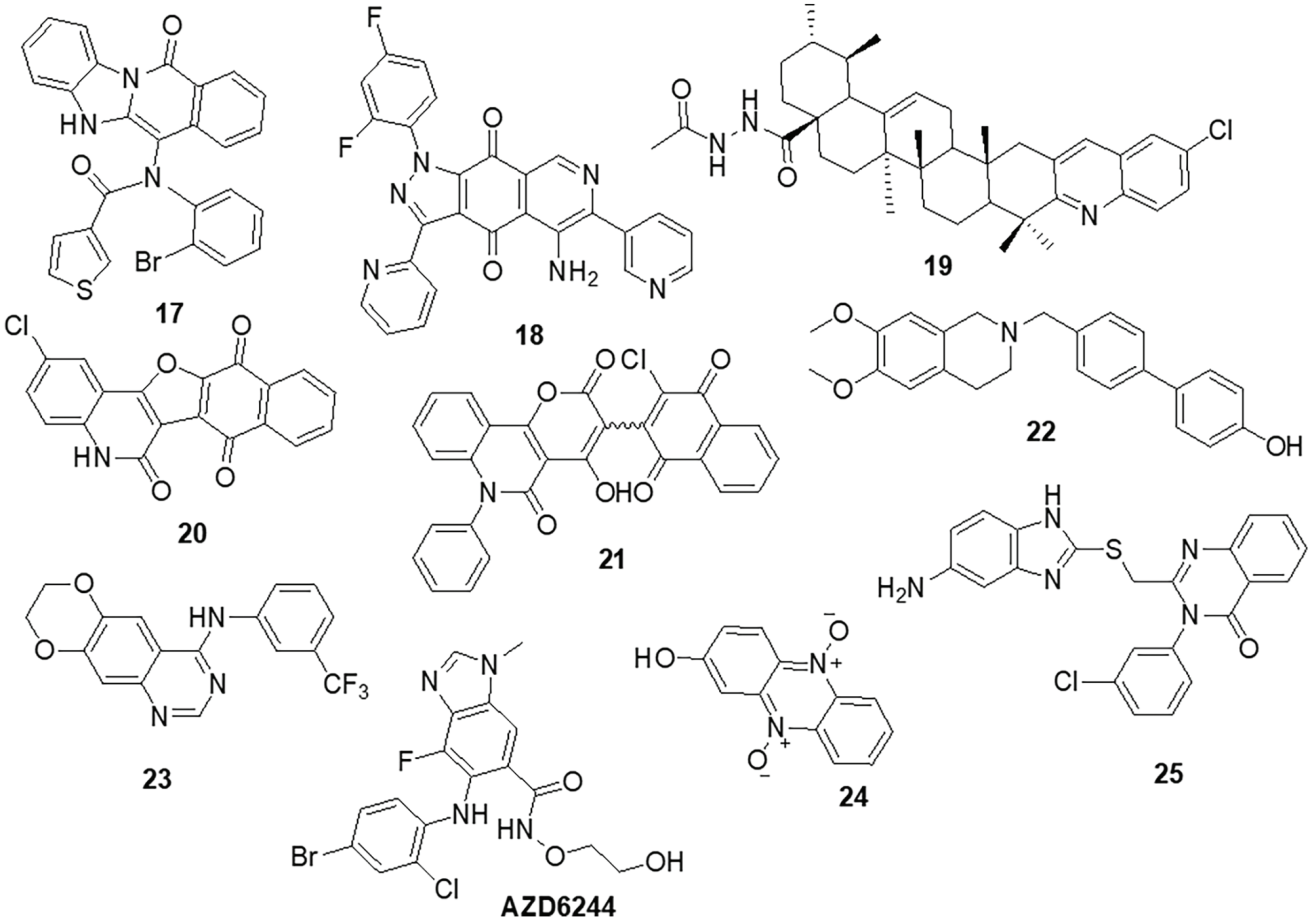
Chemical structures of quinoline, isoquinoline, quinoxaline and quinazoline containing heterocycle.

To circumvent the P-glycoprotein (Pgp) function in the brain-blood barrier (BBB) and GBM, Salaroglio et al. [128] reported novel tetrahydroisoquinoline compounds. Pgp determines resistance to a variety of GBM therapies. Pgp is a special endothelium that surrounds the brain and is abundantly expressed in GB stem cells and the BBB. The efficacy of a small set of tetrahydroisoquinoline compounds with EC_50_ of Pgp 50 nM was examined in primary human BBB cells and glioblastoma specimens obtained from patients. The higher-potency derivative **22** had an EC_50_ value of 690 nM. The compounds enhanced the number of drugs that accumulated inside neurospheres, reversing the effects of DOX-induced necrosis and apoptosis and decreasing cell viability. Three different kinds of fused tricyclic quinazolines that function as EGFR inhibitors were discovered by Chilin et al. [129]. The EGFR-overexpressing cell line A431 and the non-expressing cell line NIH-3T3 were used to evaluate the cytotoxic potential of each compound. The majority of derivatives were effective at inhibiting EGF-induced EGFR phosphorylation. The IC_50_ of compound **23** was found to be 7.10 ± 0.60 µM for NIH-3T3 and 0.77 ± 0.09 µM for A431. For the biological profile and the cytotoxic action, the size of the fused dioxygenated ring was essential. The most promising of these is the derivative **23** with the 3-trifluoromethylaniline substituent, because of its preferential binding to the inactive version of EGFR. Silva et al. [130] synthesized heterocyclic compounds and investigated the biological activity of five quinoxaline-1,4-dioxide analogs in various cell lines. Viability, migration and proliferation of malignant (B16-F10, MeWo, GL-261 and BC3H1) and non-malignant (3T3-L1 and human dermal microvascular endothelial cell) cell lines were assessed using *in vitro* cell cultures in order to assess the effects of all five derivatives. Malignant cells showed a simultaneous decrease in migration and proliferation at IC_50_ levels of all quinoxaline-1,4-di-N-oxide analogs. In all examined cell lines, compound **24** exhibited the most cytotoxic effects with an IC_50_ value of 23.4 μg/mL showed a strong anti-viability effect that was particularly noticeable in the human malignant melanocytes (MeWo) cell line. Numerous human malignancies like glioblastoma and neurodegenerative diseases like Alzheimer’s and Parkinson’s exhibit hyperactivation of the cyclin-dependent kinase (CDK)-5/p25 kinase, which is crucial for neural functioning. Since the groundbreaking discovery of the novel roscovitine, many peptide and ATP-competitive CDK5 agents have been developed. The work by Peyressatre et al. [131] brought attention to the function of quinazolinone derivatives in CDK5 targeting at a site other than the ATP pocket. This approach made them promising leads for the treatment of glioblastoma as well as neurological illnesses. These substances offered potential alternatives for the drawbacks of traditional ATP-competitive inhibitors that targeted the CDK5/p25 interface. In kinase and cell proliferation studies, compound **25** showed the highest relative inhibitory potential of all the compounds, making it the most promising candidate for a treatment. *In vitro* kinase inhibition experiments indicated the specificity of compounds towards the target of interest (Fig. 4).

### Thiazole and thiadiazole containing heterocycles

Szeliga et al. [132] reported 1,3,4-thiadiazole derivatives as an anticancer agent. All thiadiazole derivatives exhibited antitumor action in both patient-derived GBM cells and human GBM cell lines. The IC_50_ values for the investigated drugs were 15–110 folds less than those for standard chemotherapeutic treatment, TMZ. Compound **26** appeared to be the most promising candidate for the medications under study, as it demonstrated IC_50_ values ranging from 45 to 68 μM for GBM cells and >100 μM for human astrocytes. The action of TMZ was markedly increased by compound **26**, which also prevented GBM cells from proliferation and migration. Additionally, it reduced the degree of GSK3 and AKT phosphorylation, which in part aided in the suppression of cell survival. The study by Finiuk et al. [133] set out to compare the antiproliferative and proapoptotic effects of thiazole derivatives to those of the positive controls, TMZ and DOX, in glioma cells. The anticancer effects of molecules **27**, TMZ and DOX on U-251 and T98-G cells were assessed using the MTT assay. Western blot analysis, fluorescence microscopy, flow cytometric analysis, the agarose gel retardation assay and the DNA comet assay in an alkaline media were used to examine the effect of molecule **27** on U-251 cells. This compound demonstrated approximately a 20-fold increase in cytotoxicity towards U-251 with an IC_50_ of 10.8 ± 0.98 μM and T98-G cells with an IC_50_ of 12.6 ± 1.35 μM, as well as a two-fold increase in activity, when compared to the effects of TMZ. In U-251 cells, compound **27** induced apoptosis by activating caspase 3 and PARP1. It also caused an elevation in the levels of the proapoptotic proteins Bax and Bim and a reduced amount of phosho-ERK1/2 kinase. Compound **27** was responsible for a significant increase in the percentage of cells in the G2 phase. Responses by TMZ and DOX were shown to have caused similar changes. Additionally, TMZ and DOX enhanced the prercentage of S-phase cells. Compound **27**, TMZ and DOX caused an increase in the several pre-G1 phase (apoptotic) cells. As a result, compound **27** was suggested to arrest cells in the G2/M phase. Lee et al. [134] designed anthra [1,2-c] [1,2,5] thiadiazole-6,11-diones and examined them for cell proliferation, apoptosis and signaling pathways. The most effective derivative **28** caused cell death in DU-145 tumor cells along with a reduction in the activity of the ERK1/2 and p-38 signaling cascades. Compound **28** exhibited strong efficacy against the DU-145 cell line, with an IC_50_ of 4.53 μM. All screened molecules showed cytotoxic and cytostatic actions, suggesting their use in generating additional anticancer medicines. Furthermore, in an effort to target cancer, Charitos et al. [135] designed, synthesized and tested some novel 3,6-disubstituted 1,2,4-triazolo-[3,4-b]-1,3,4-thiadiazole analogs. Recently synthesized derivatives, especially **29** and **30**, have shown significant *in vitro* cytostatic and cytotoxic anti-tumor activity with IC_50_ >100 μM. Additionally, they have shown relatively low acute toxicities *in vivo*, indicating the possibility of high therapeutic ratios. Numerous protein targets have been identified through *in silico* screening, such as apoptotic protease-activating factor 1 (APAF1) and tyrosine-protein kinase HCK. These targets may be connected to the bioactivities of active derivatives in ovarian, epithelial ovarian cancer and adenocarcinoma cell lines.

**Figure 5:**
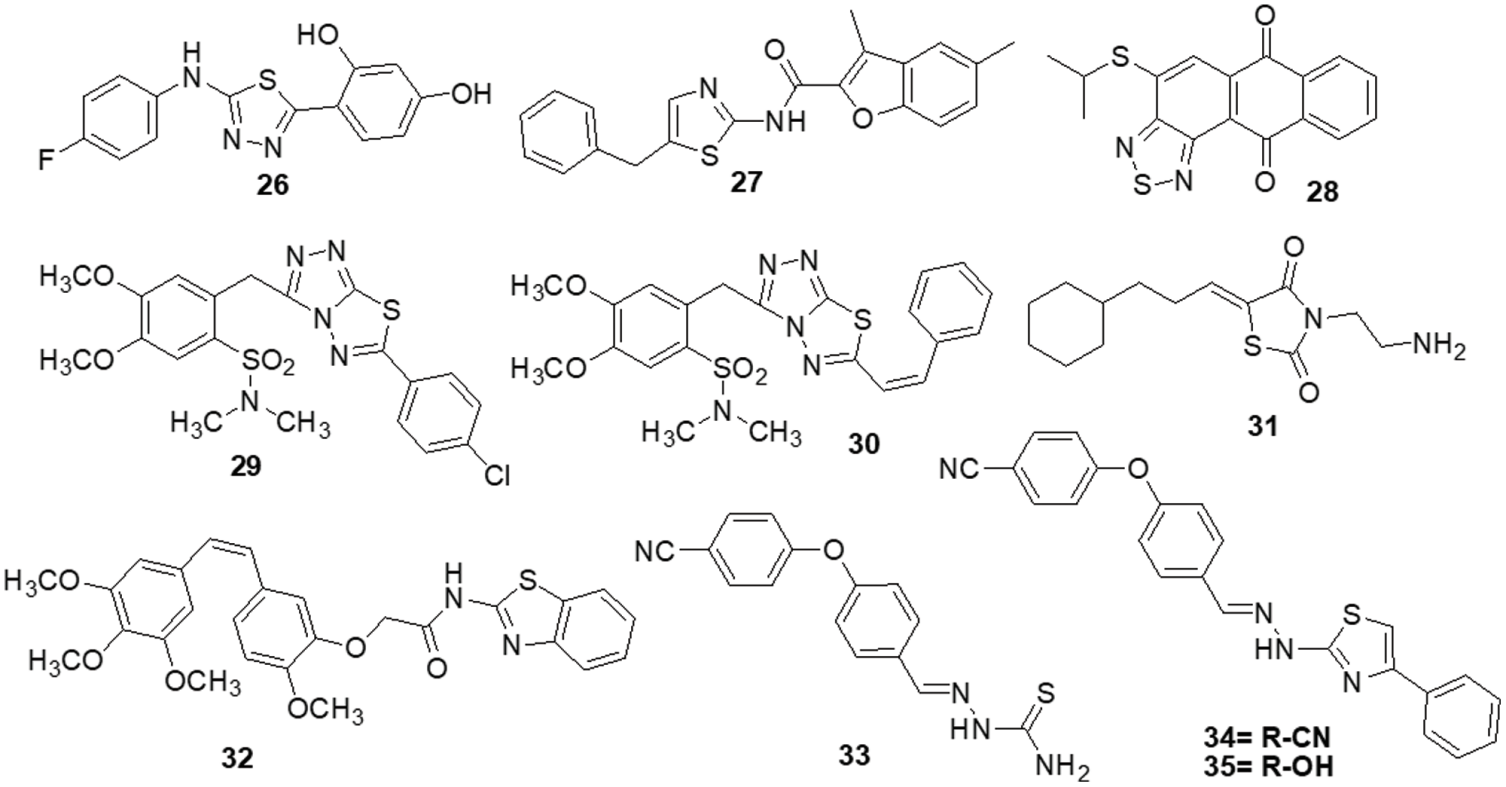
Chemical structures of thiazole and thiadiazol containing heterocycle.

Synthesis and pharmacological assessment of a series of 2,5-disubstituted thiazolidine-2,4-dione analogs were reported by Liu et al. [136]. They discovered anticancer activity that may function by inhibiting the RAF/MEK/ERK and PI3K/AKT signaling pathways. A new compound **31** was found to have improved anticancer activities in U-937, M-12 and DU-145 cancer cells. Compound **31** was found to have an IC_50_ of 3.4 ± 0.043 μM for the U937 cell line. Additionally, it has been observed to stop U937 cells in their S phase. Furthermore, western blot analysis demonstrated a correlation between anti-proliferative activity and inhibition of the RAF/MEK/ERK and PI3K/AKT signaling pathways. These results encouraged the further development of **31** as a new lead with multi-target properties to produce anticancer drugs that are more potent. Several combretastatin-amidobenzothiazole conjugates were synthesized by Kamal et al. [137] and their anticancer effectiveness was assessed. Being the strongest derivative, compound **32** reported GI_50_ values ranging from 0.019 to 11 μM against cancer. Biological studies of tubulin polymerization, cell cycle distribution and the ERK signaling system were conducted using the MCF-7 cell line. The results of a FACS study showed that these substances caused arrest at the G2/M phase of the cell cycle. Molecule **32** significantly reduced the amounts of ERK1/2, p-ERK and c-Jun proteins by affecting the ERK signaling pathway and tubulin polymerization. Docking tests have demonstrated that the active compounds engage and interact well in the ATP binding domain of ERK protein. Altıntop et al. [138] synthesized new thiazole compounds and tested them for their cytotoxic activities against AKT kinase-targeting cells A-549, C6 and NIH-3T3. Due to its particular inhibitory effects on C6 and A-549 cells, compound **33** was shown to be the most effective anticancer medication, with IC_50_ values of 3.83 ± 0.76 µg/mL and 12.0 ± 1.73 µg/mL, respectively. Additionally, molecule **33** strongly inhibited the AKT enzyme and increased the number of early and late apoptotic cells in the C6 cell line (32.8%) compared to cisplatin (28.8%). Molecular docking study was done to predict possible binding modes for molecules **33**, **34** and **35** inside the AKT active region (PDB code: 4EJN). The biological activity was found to be supported by molecular docking simulations since they agreed with *in vitro* experiments (Fig. 5).

### Imidazole containing heterocycles

Dos Santos et al. [139] reported imidazo [1,2-a] pyridine compounds, which have been extensively employed in drug design and development. Compound **36** showed potential activity against A172 cells with an IC_50_ of 1.8 μM and resulted in selectivity for this glioblastoma cell. Additionally, the research suggests that compound **36** may facilitate cell cycle arrest caused by the suppression of the AKT/mTOR/HIF-1 pathway. Compound **36** has also demonstrated efficacy in downregulating the members of the MAPK family of proteins. It also inhibited MEK/ERK1/2 signaling and showed an antiproliferative effect. Furthermore, it inhibited p38 MAPK, which exhibited an anti-inflammatory effect by inhibiting inflammasome complex proteins like NLRP3 and caspase-1 present in glioblastoma cells. SFKs have attracted a lot of attention as promising targets for GBM treatment. Zhang et al. [140] discovered a novel family of imidazo [4,5-c] pyridine-2-one compounds that function as SFK inhibitors. Comparable to lead compound **37**, compound **38** demonstrated effective action against T-98G, U-87, U-251 and U-87-EGFRvIII GBM cell lines with IC_50_s of 16.39 ± 2.12, 30.30 ± 0.84, 24.61 ± 2.28 and 50.46 ± 0.97 μM, respectively. Using molecular dynamics (MDs) modelling, the most active molecule **38** is found to be bonded to the ATP binding site of SFKs in multiple ways. Compound **38** satisfies the criteria for treatments affecting the central nervous system (CNS) based on its ADME prediction. These findings prompted us to choose a new SFK inhibitor as a potential GBM treatment. Mutations in the DNA lead malignant gliomas to be immune to cell death, which makes them very resistant to current treatment approaches. Increased cell proliferation and resistance to apoptosis are facilitated by S/TK and casein kinase 2 (CK2), which is often constitutively active and overexpressed in malignancies. Kaminska et al. [141] discovered a benzimidazole derivative in their study as a CK2 inhibitor. The CK2 inhibitor compound **39** was found to be capable of minimizing cell survival by 50% in C6 GBM cells at a dose of 50 μM. Compound **39** plays a major role in apoptosis by activating caspases 3 and 7, which is followed by the cleavage of poly (ADP-ribose) polymerases (PARP). Different cancer cells were influenced by CK2 expression inhibition or therapy with CK2 inhibitors, which reduced survival or triggered apoptosis.

**Figure 6:**
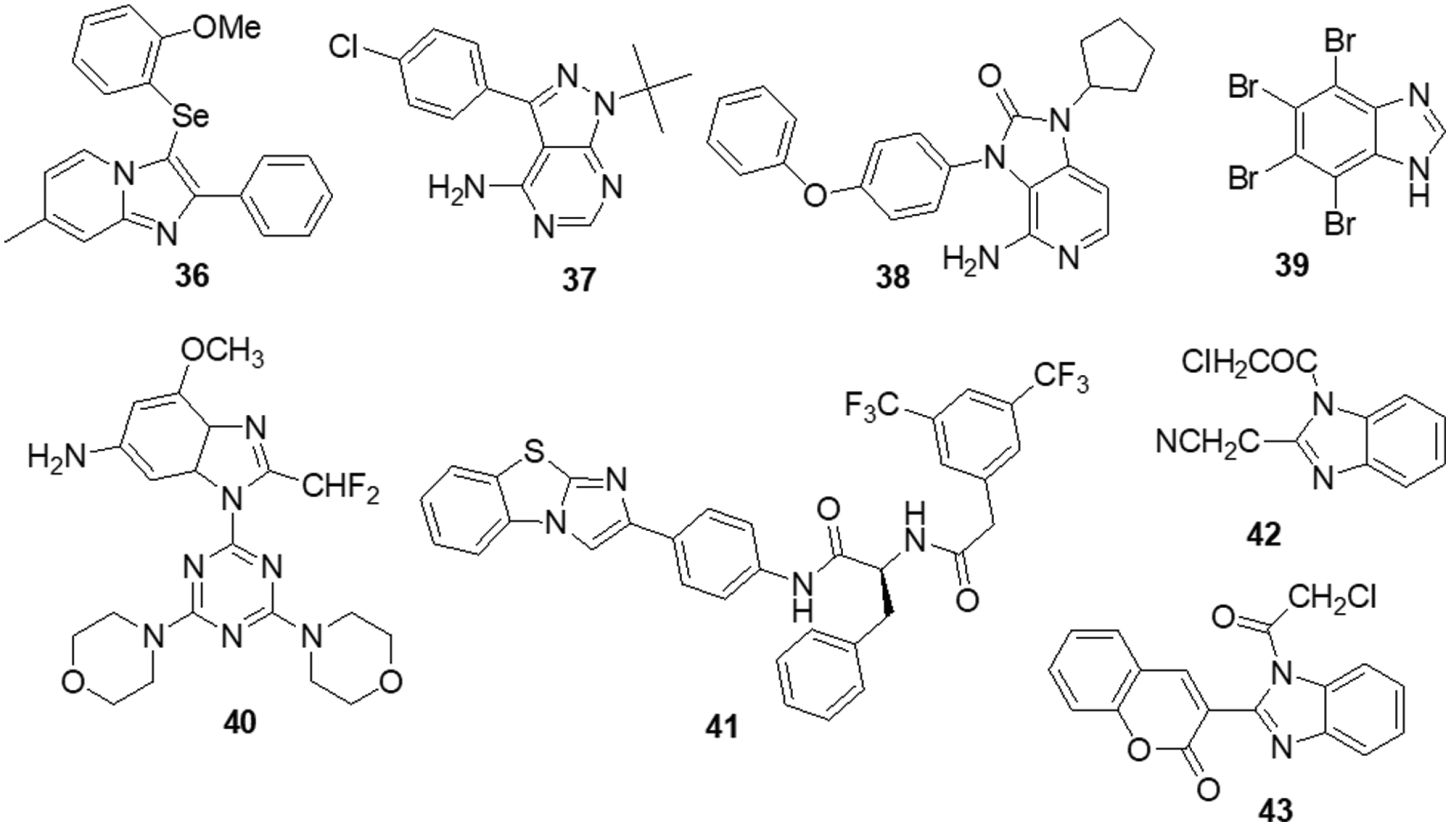
Chemical structures of imidazole containing heterocycle.

To increase the potency of a pan-class I PI3K inhibitor, Rewcastle et al. [142] found benzimidazole rings modified at the 4 and 6 positions. The 6-amino-4-methoxy **40** analog significantly inhibited the class Ia PI3K enzymes (p110α, p110β and p110δ) against all three. It was also significantly potent against two mutated versions of the p110α isoform (E545K and H1047R). Additionally, the compound was also evaluated *in vivo* in Rag1 mice using a U-87MG human glioblastoma tumor xenograft model. At a dosage of 50 mg/kg administered through IP injection on a qd 10 dosing schedule, it significantly decreased tumor development by 81% in comparison with controls. The Met RTK is a prospective target for anticancer treatments because of its role in tumor formation and treatment resistance. Here, Furlan et al. [143] revealed the discovery of bioactive 4-(imidazo [2,1-b] benzothiazol-2-yl) phenyl analogs that target cancer cells dependent on oncogenic Met. One of these derivatives, molecule **41**, inhibited *in vivo* carcinogenesis, anchorage-independent growth and survival without causing any negative side effects. The IC_50_ value of molecule **41** for Met in this cellular system was 4 μM and that was higher than the IC_50_ value assessed in HepG-2 and GTL-16 cells. A novel benzimidazole sequence was studied by Milad Mohareb et al. [144]. Compounds **42** and **43** were tested with foretinib used as the standard reference, as researchers assessed the ability of the newly synthesized compound to inhibit *in vitro* growth in terms of IC_50_ against six tumor cell lines: A-549, MKN-45, H-460, U-87MG, HT-29 and SMMC-7721. The findings indicated that the majority of compounds were significantly active against the selected cell lines (Fig. 6).

### Indole containing heterocycles

**Figure 7:**
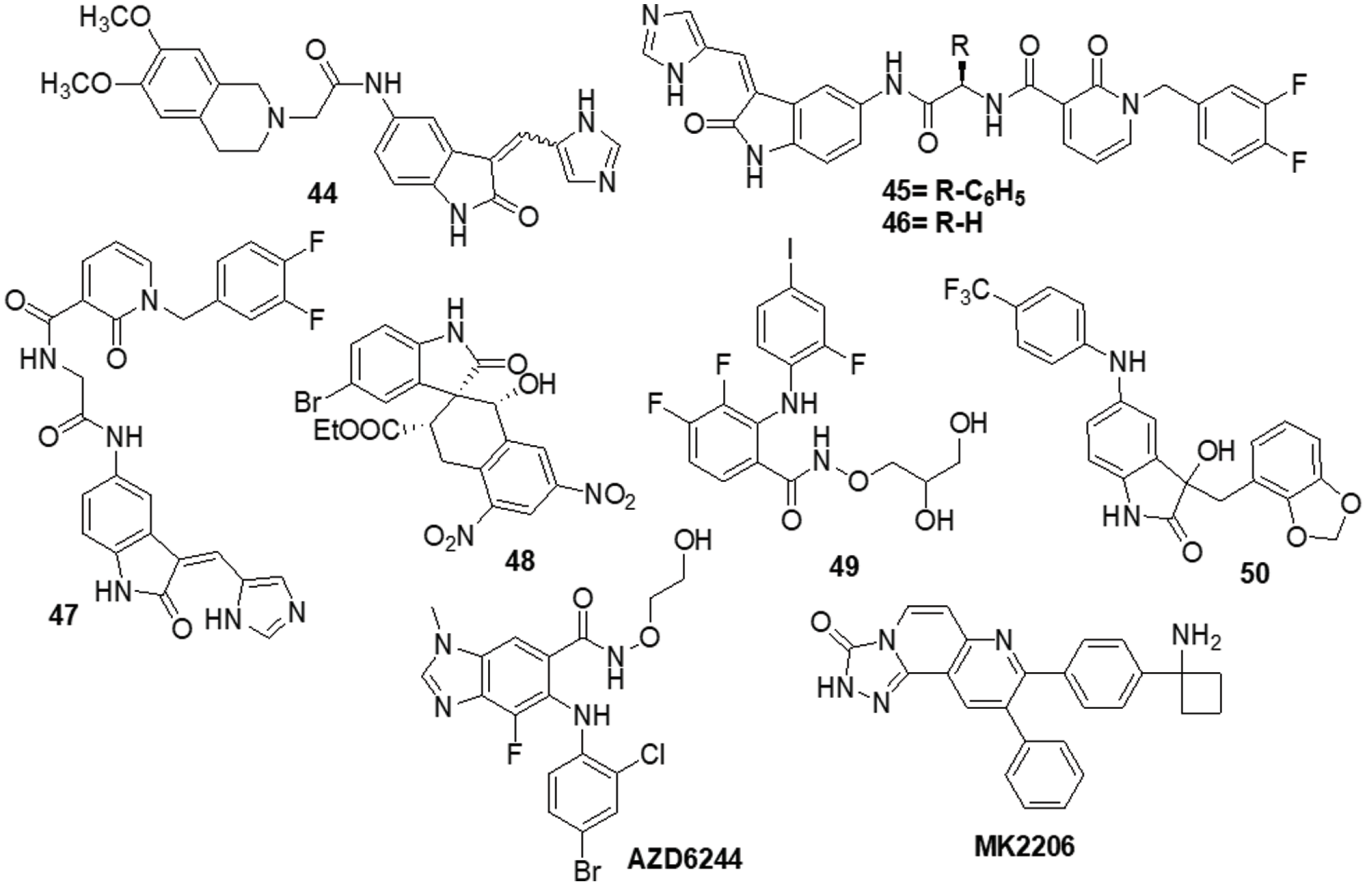
Chemical structures of indole containing heterocycle.

The various signaling pathways involved in maintaining the GBM-derived stem cells (GSCs) stemness, mitogenic capacity and anti-apoptotic properties which make the PDK1/AKT pathway a challenging target for the development of innovative potential medications able to alter GBM resistance to chemotherapy. Sestito et al. [145] developed a small family of 2-oxindole derivatives through the rational design and synthesis of novel PDK1/AKT inhibitors. Compound **44** suppressed downstream effectors important in GCS survival, including as CHK1,GS3Kα and GS3Kβ, as well as PDK1 kinase. The findings indicated that compound **44** inhibited PDK1 with an IC_50_ of 980 nM. Compound **44** was used as a model for multi-target therapy for GBM and seemed to be a useful method for examining the involvement of the PDK1/AKT mechanism in GCS self-renewal and tumor progression. Casari et al. [146] identified novel 2-oxo-indole derivatives in a panel of pancreatic cancer cell lines and examined them for dual inhibitory effect towards PDK1 and Aurora kinase A. In the PDK1 inhibition experiment, compounds **45** and **46** displayed IC_50_ values of 0.11 and 0.41 μM, respectively. Molecule **45** exhibited an IC_50_ of 0.44 μM for U-87MG cells and an IC_50_ of 0.003 μM for GSC growth. On the other hand, it has been shown that compound **46** inhibits Aurora Kinase A and PDK1. This compound was capable of inducing cell death in U-87MG cells, which decreased cell growth and proliferation. Furthermore, compound **46** demonstrated an IC_50_ of 0.008 μM for hindering the production and growth of GSC extracted from U-87MG cells. Similar results were obtained when GSC was derived from the U-343MG and ANGM-CSS cells, with IC_50_ values of 0.05 and 0.04 μM, respectively. PDK1 is a crucial component of the PI3K/AKT pathway. Sestito et al. [147] produced a unique series of molecules by combining the 2-oxo-indole scaffold with the 2-oxo-pyridonyl fragment. Several compounds were synthesized to understand their manner of binding, docking and molecular dynamics (MD) in tandem. *In vitro* experiments were carried out on the compounds to assess their anticancer potential and lowest IC_50_. OXID-pyridonyl hybrid **47** was tested on kinases associated with the PI3K/PDK1/AKT pathways and it was found to have an IC_50_ of 112 nM. Together, the findings showed that compound **47** is the potent molecule of a new class of PDK1 blockers. Wang et al. [148] synthesized several chiral spirocyclic tetrahydronaphthalene (THN)-oxindole hybrids and evaluated them against MDM2 and CDK4 together for the treatment of glioblastoma. The *in vitro* experiment demonstrated a 40-fold difference in selectivity between the lowest and most effective stereoisomers. Inhibition of MDM2-P53 interaction and CDK4 activation was observed in glioblastoma cell lines by **48**, which caused apoptosis and cell cycle arrest. The cell cycle pathway was up-regulated by P53 and related proteins in **48**-treated cells. Mouse glioblastoma xenografts that were effectively treated with anti-tumor activity showed effective treatment. The discovery of new tetrahydronaphthalene-linked spirooxindoles resulted in MDM2-CDK4 dual inhibitors that were effective in treating glioblastoma, as evidenced by these findings. Compound **48** had an inhibitory concentration (IC_50_) of 4.9 ± 0.5, 8.6 ± 0.6 and 9.5 ± 0.7 μM against the U-87MG, U-251 and T98-G cell lines, respectively. Yu et al. [149] synthesized a new generation of indolone derivatives that have shown dual activity against the RAF/MEK/ERK and PI3K/PDK1/AKT pathways. The binding pattern in the MEK allosteric pocket is replicated by synthesized compound **49** and 3-substituted indolone maintains its inhibition of PDK1 similar to that of BX517. When the compounds were tested against the cancer cell lines A-549 and H460, molecule **50** was found to be highly effective with an IC_50_ value of 1.8 ± 0.8 μM. The mechanistic study on the RAF/MEK/ERK and PI3K/PDK1/AKT pathways was conducted by conducting mechanistic studies on this molecule with the usage of antiphosphorylation activity assays, with AZD6244 and MK2206 being positive controls. When the molecule was present at higher concentrations, like 2.5 μM, both routes were inhibited. It has been determined via docking studies that the hydrogen bond formed between the hydroxy group and Met219 residue is the key factor behind its anticancer properties (Fig. 7).

### Acridone containing heterocycles

**Figure 8:**
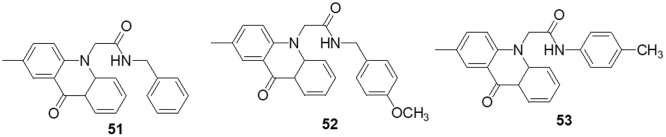
Chemical structures of acridone containing heterocycle.

Singh et al. [150] reported the use of acridone (9-oxo-9,10-dihydroacridine-4-carboxylic acid) derivatives as potent antiproliferative medications. The MTT assay, phase contrast micrographs and confocal pictures of immunostained C6 glioma cells for biomarkers including α-tubulin, GFAP, mortalin and HSP-70 cells all showed that all the derivatives had considerable anticancer action. These molecules had IC_50_ values that ranged from 14 to 20 μM. According to flow cytometry data, the molecules slowed down the cell cycle during the G0/G1 phase. The overexpression of microtubule affinity-regulating kinase 4 (MARK4) is a common cause of malignancies, making it a special target for antitumor action. Voura et al. [151] described various acridone compounds and synthesized, characterized and tested them for inhibitory action against human MARK4 in their search for novel, efficient MARK4 inhibitors. As determined by fluorescence binding, ITC and kinase assays, three of the synthesized compounds (**51**, **52** and **53**) were reported to possess greater binding affinity as well as enzyme inhibitory activity in the μM concentrations. A panel of 26 kinases from the same family that were commercially accessible was used for functional testing of selected putative lead compounds. The kinase selectivity profile of each derivatives was unique. Submicromolar cellular action against MARK4 has been observed by certain molecules that have been discovered. Furthermore, when compounds **51**, **52** and **53** were tested *in vitro* against malignant cell lines (MCF-7 and HepG-2), it was discovered that they inhibited cell growth and mainly promoted cell death in MCF-7 cells, with IC_50_ values of 5.2 ± 1.2, 6.3 ± 1.2 and 5.8 ± 1.4 μM, respectively (Fig. 8).

### Triazine containing heterocycles

**Figure 9:**
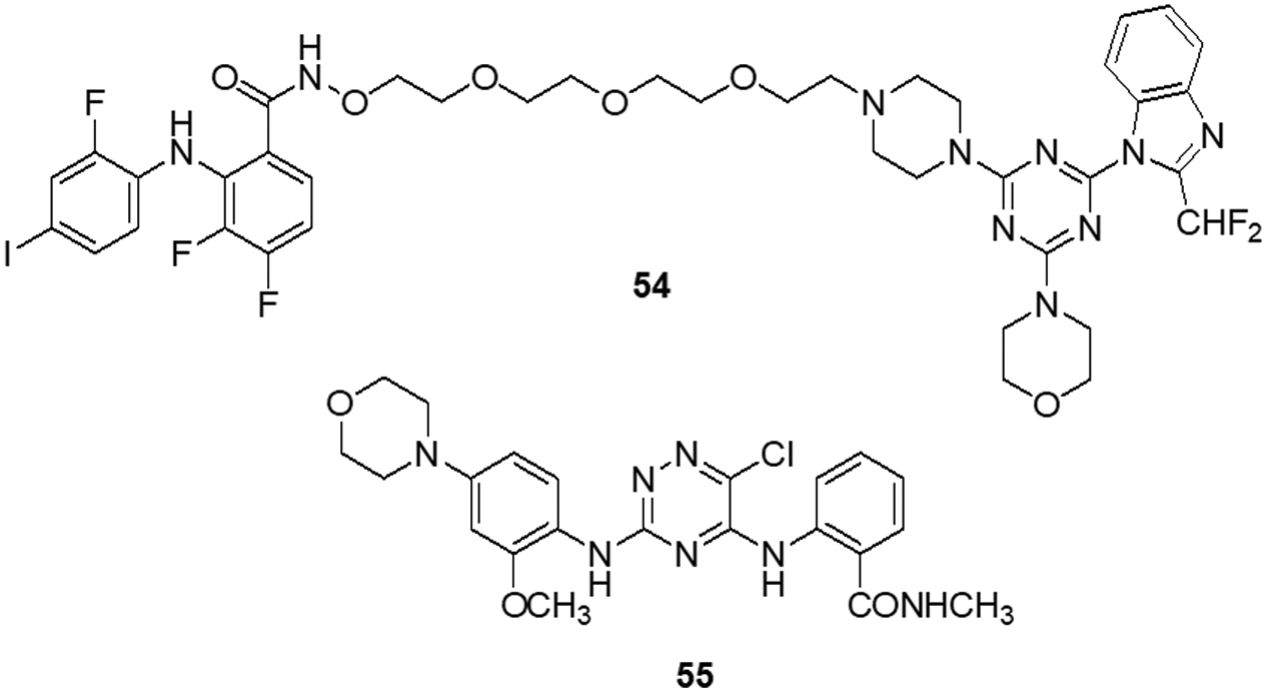
Chemical structures of triazine containing heterocycle.

Van Dort et al. [152] revealed the covalent linkage of molecular analogs of the ATP-competitive PI3K and the ATP-non-competitive MEK inhibitors to produce a series of single-entity and bifunctional MEK1/PI3K inhibitors. *In vitro* enzymatic inhibition experiments revealed that inhibitors strongly inhibited both PI3K (IC_50_ = 54–341 nM) and MEK1 (IC_50_ = 0.015–56.7 nM). It was demonstrated that inhibitor **54** concurrently inhibited PI3K and MEK1 in two tumor cell lines (A-549 and D54). Like the combination administration of equal dosages of standard drugs, inhibitors cause a dose-dependent decrease in cell viability. **54** proved beneficial *in vivo* following oral dosage in a mouse model of D54 glioma tumor. Western blot analysis of compound **54** at 2 h after delivery revealed a 95% and 67% reduction in tumor ERK1/2 and AKT phosphorylation, respectively. The outcome proved the bioavailability and potency of this dual inhibitor strategy for combined MEK1/PI3K inhibition. Recent research has demonstrated that a group of FAK inhibitors that are 1,3,5-triazine have antiangiogenic and antitumor activity against HUVEC cells and cancer cell lines, respectively. As a novel framework for FAK inhibitors, Dao et al. [153] created and synthesized several unique compounds with a 1,2,4-triazine core. The best compound, **55** with an IC_50_ value of 0.23 μM against the enzyme FAK, was one of the compounds that revealed 10^–7^ M IC_50_ values. Among these, some inhibitors efficiently inhibited the proliferation of the glioblastoma cell line U-87MG. Compound **55** was docked within the FAK kinase active site in order to examine its potential binding mechanism. The ATP-binding pocket easily accommodates compound **55 **(Fig. 9).

### Other heterocycles

According to Da Silva et al. [154] a novel class of 1,4-disubstituted 1,2,3-triazole compounds was developed and reported inhibition against several resistant glioblastoma cell lines, GBM-02 and GBM-95, was assessed. Compound **56** was found to be highly efficient and possess the tosyl-hydrazone group and the triazole-linked methylenoxy moiety. Compound **56** was shown to have IC_50_ values of 27.1 and 28.7 μM against the GBM-95 and U87 cell lines, respectively. An X-ray diffraction examination of a single crystal was used to investigate the structure of this chemical. Dual-specificity tyrosine phosphorylation-related kinase 1A (DYRK1A) is a protein kinase that catalyses autophosphorylation as well as phosphorylation. Especially in glioblastoma, DYRK1A expressed higher than other cancer. New heterocyclic diphenolic compounds were synthesized and their biological effects as novel DYRK1A inhibitors were reported by Zhou et al. [155]. The structure of lead drug **57** modification served as the basis for the production of several heterocycles, including benzimidazole, imidazole, naphthyridine, bipyridine, pyrazole-pyridines and triazolopyrazines, which were then tested for their ability to inhibit DYRK1A. Among the heterocyclic scaffolds, the 7-azaindole scaffold was found to be more effective against DYRK1A than the others. A comparison of compound **58** with the lead molecule **57** suggested that analog **58** was the most potent bioactive molecule among this novel group of compounds. Ciftci et al. [156] revealed a variety of pentacyclic triterpene analogs were put through *in vitro* as well as *in silico* experiments and evaluated their effectiveness in targeting EGFR. Compound **59** was the most effective anti-glioma drug, with an IC_50_ value of 5.82 μM for U-251 human glioblastoma cells. Compound **59** exhibited modest cytotoxicity to peripheral blood mononuclear cells (PBMCs) and selectively inhibited Jurkat human leukemic T-cell growth. Moreover, this compound inhibited EGFR with an IC_50_ value of 9.43 μM and also promoted cell death in comparison to erlotinib (IC_50_ = 0.06 μM). Compound **59** was a promising orally accessible anti-glioma drug that targeted EGFR and could pass through the BBB. Mohareb et al. [157] reported that dimedone and aryl aldehydes interacted to produce derivatives of benzylidene. These then went through an array of heterocyclization processes to form derivatives of pyrazole, isoxazole, thiophene and pyridazine. The drug’s efficacy against different tumor cell lines was evaluated using inhibitors of tyrosine kinases and Pim-1 kinases. The inhibitory activity of all produced compounds against the cancer cell lines A-549, H460, HT-29 and MKN-45 was tested using an MTT assay, with foretinib serving as the positive control. The potential compounds displayed an IC_50_ against tyrosine kinase and Pim-1 kinase in the micromolar range. To assess the potential effectiveness of the reported mTOR kinase inhibitors. Gini et al. [158] performed *in vitro* and *in vivo* investigations in glioblastoma cells and an intracranial model. It has been observed that compound **60** inhibits rapamycin resistance signaling and stops the growth of GBM. The outcome shown that compound **60** effectively inhibits mTORC2 signaling, decreases rapamycin-resistant mTORC1 signaling and slows the growth of glioblastomas both *in vitro* and *in vivo*. PTEN loss and EGFRvIII expression increase compound **60** sensitivity, which is consistent with increased efficacy in cancers with significant mTOR activation. Additionally, compound **60** successfully induces autophagy, delaying the death of tumor cells. GBM cells and orthotopic xenografts are very susceptible to molecule **60** induced cell death when autophagy is genetically or pharmaceutically inhibited. These results suggest that mTOR kinase inhibitor **60** may be useful in the treatment of GBM and provide a way to identify the patients who will benefit most from mTOR inhibition. Guerra et al. [159] reported benzofuran derivatives, among them compound **61**, was a potent and selective inhibitor of CK2 and S/TK that impacted the signaling pathway. Compound **61** showed an IC_50_ of 40 μM after 48 h in M059K cells. By using ELISA, FACS and Western blot-based assays, it was determined that cancer cells had altered cell viability, cell death and impacts on the main signal transduction pathways. Treatment with compound **61** induced C-caspase-mediated apoptotic cell death together with suppression of EGFR expression and suppression of NF-κB transcriptional activity.

**Figure 10:**
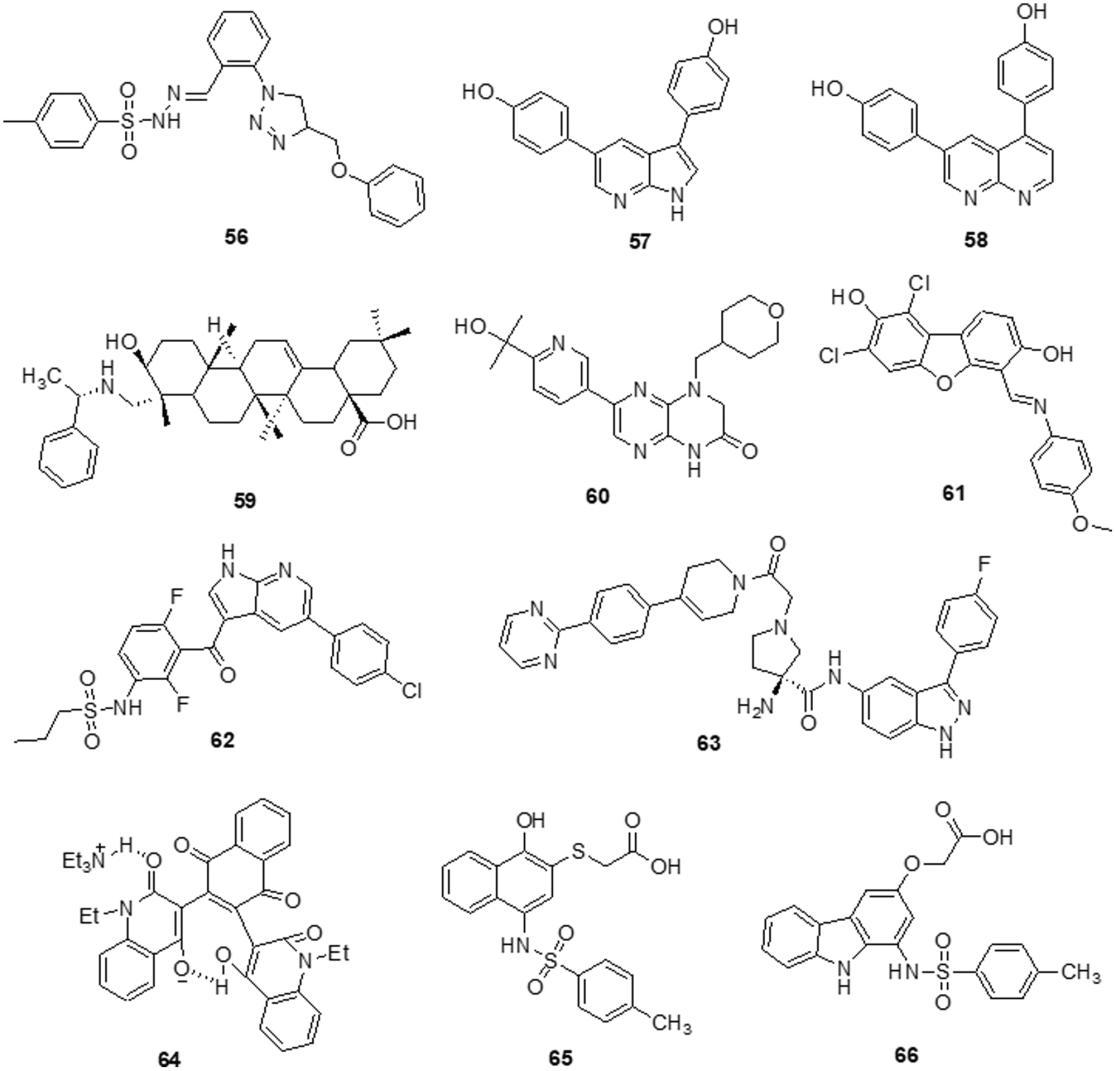
Chemical structures of other heterocycle against glioma.

The protein kinase BRAF is the one that is most frequently altered in human malignancies. It was discovered that melanoma tumors frequently carry oncogenic mutations in the BRAF gene and that these tumors depend on the RAF/MEK/ERK pathway. There was hope that blocking BRAF kinase activity may benefit melanoma patients. Bollag et al. [160] discovered compound **62** as a strong inhibitor of oncogenic BRAF kinase activity. Preclinical studies indicates that **62** caused BRAF mutant xenografts to grow regressively by preferentially inhibiting the RAF/MEK/ERK pathway in BRAF mutated cells. Boga et al. [161] synthesized 3-substituted pyrrolidine derivatives to enhance the PK characteristics of an inhibitor of ERK1/2. Extensive lead optimization resulted in the finding of 3-thiomethyl pyrrolidine and improved AUC values in rats at a dose of 10 mph *per os* (PO) by 10–14 times. ERK showed strong enzymatic activity towards compound **63** with an IC_50_ of 7 nM. In rats, it had a total clearance of 8.4 ml/min/kg, a half-life of 2.5 h and a 70% bioavailability. Several novel compounds with a bis-quinolinyl naphtha quinone moiety were synthesized by Aly et al. [162]. The drugs were tested against various tumor cell lines as well as kinases, including ERK2 and JNK2. Additionally, molecular docking studies against the ATP-binding site of ERK2 were performed to display the binding pattern of the drugs (PDB: 4O6E). The results demonstrated that compound **64** had an exceptional IC_50_ value of 0.71 µM for ERK2 inhibition. Compound **64** was primarily responsible for suppression of Ets-1 phosphorylation by ERK2. Xi et al. [163] described the use of virtual screening and pharmacophore modeling to identify novel allosteric MEK inhibitors. Using structural derivations from the successful drug **65** (IC_50_ = 27.2 ± 4.5 µM), bioisosterism substitution and substituent modification were used to create a limited library of carbazoles. Enzymatic experiments revealed the initial structure-activity relationships and the derivative **66** (IC_50_ = 12.8 ± 0.5 µM) significantly inhibited the RAF-MEK cascade. Single or multitarget kinase inhibitors against the MEK/ERK and PI3K/AKT pathways may be advantageous from a therapeutic standpoint (Fig. 10) (Table 1).

**Table 1:** IC_50_ values for enzyme inhibition of novel compounds

Heterocycles targeting protein kinases	Novel compounds	Cell lines tested	IC_50_ values	Targeting kinases/pathways	References
**Pyrimidine containing heterocycles**	Molecule 1	DBTRG.05-MG	61.8 ± 0.9 μM	EGFR kinases	[111]
	Molecule 2		41.2 ± 1.2 μM		
	Molecule 3	MCF-7	9.29 μM	Topoisomerase II	[112]
			0.2 μM	VEGFR-2	
	Molecule 4	MCF-7	20.22 ± 1.50 µM	FASTK	[113]
	Molecule 5		6.52 ± 0.82 µM		
	Molecule 6		8.20 ± 0.61 µM		
	Molecule 7	U-87	5.5 ± 0.4 nM	FAK	[114]
	Molecule 8	A-549	0.27 µM	FAK	[115]
		U-87MG	0.16 µM		
		MDA-MB-231	0.19 µM		
	Molecule 9	U-87	0.035 ± 0.017 µM	PI3K/AKT	[116]
	Molecule 10	H460	7.43 ± 1.45 μM	PI3K/mTOR	[118]
		PC-3	11.90 ± 0.94 μM		
	Molecule 11	A-549	10.5 ± 0.75 μM	mTOR kinase	[119]
		PC-3	14.4 ± 1.1 μM	PI3Kα kinase	
		MCF-7	11.8 ± 0.69 μM		
		HeLa	10.3 ± 0.58 μM		
		HepG-2	8.77 ± 0.83 μM		
	Molecule 12	U-87	7.1 ± 0.16 μM	SFK	[120]
	Molecule 13	C2C12	24.2 μM	SFK	[121]
	Molecule 14	PC-3	7.71 µM	ERK2	[122]
		Hep-2	5.40 µM		
		HeLa	4.30 µM		
	Molecule 15	MCF-7	1.937 μM	ERK2	[123]
		HepG-2	3.695 μM		
		HCT-116	4.134 μM		
	Molecule 16	MDA-MB-231	20 μM	Caspase 8, Caspase 9, PARP1, Bax, Bcl-2, p21 and p27	[124]
		HT-29			
		U-87MG			
**Quinoline, isoquinoline, quinoxaline and quinazoline containing heterocycles**	Molecule 17	U-87	24.9 µM	PI3K/AK, RAF/MEK/ERK	[78]
		LN-229	27.1 µM		
	Molecule 18	U-251	<2.5 µM	PI3K/mTOR	[125]
		DBTRG			
		U-87MG			
	Molecule 19	MDA-MB-231	0.12 ± 0.01 µΜ	RAS/RAF/MEK/ERK	[126]
		SMMC-7721	0.34 ± 0.03 µM		
		HeLa	0.08 ± 0.01 μM		
	Molecule 20	A-375	3.7 µM	ERK1/2	[127]
	Molecule 21		0.13 µM		
	Molecule 22	BBB Cells	690 nM*	Pgp	[128]
	Molecule 23	A431	0.77 ± 0.09 µM	EGFR	[129]
	Molecule 24	MeWo	23.4 μM	PI3K/AKT/ NF-κB	[130]
	Molecule 25	U-87	7.6 ± 3.8 µM	CDK5/p25	[131]
**Thiazole and thiadiazole containing heterocycles**	Molecule 26	T98G	53.53 ± 4.77 µM	c-Met/ FAK/ AKT	[132]
		U-87MG	44.98 ± 9.71 µM		
		LN229	55.20 ± 9.07 µM		
	Molecule 27	U-251	10.8 ± 0.98 μM	Caspase 3, PARP1	[133]
		T98-G	12.6 ± 1.35 μM		
	Molecule 28	DU-145	4.53 μM	ERK1/2, p-38	[134]
	Molecule 29	SKOV-3	>100 μM	APAF1, HCK, CDK2, MMP3	[135]
	Molecule 30	OVCAR-3			
		MCF-7			
		T-47D			
		PC-3			
		A-431			
	Molecule 31	U-937	3.4 ± 0.043 μM	RAF/MEK/ERK, PI3K/AKT	[136]
	Molecule 32	MCF-7	0.019–11 μM	ERK1/2	[137]
	Molecule 33	C6	3.83 ± 0.76 µM	AKT	[138]
	Molecule 34		3.83 ± 0.76 µM		
	Molecule 35		5.83 ± 0.76 µM		
		A-549	12.0 ± 1.73 µM		
			12 ± 1.73 µM		
			106.67 ± 5.77 µM		
**Imidazole containing heterocycles**	Molecule 36	A172	1.8 μM	AKT/mTOR/HIF-1, MEK/ERK1/2	[139]
	Molecule 37	T-98G	12.52 ± 2.37 μM	SFK	[140]
	Molecule 38		16.39 ± 2.12 μM		
		U-87	39.94 ± 6.91 μM		
			30.30 ± 0.84 μM		
		U-251	21.14 ± 2.16 μM		
			24.61 ± 2.28 μM		
		U-87-EGFRvIII	49.11 ± 7.76 μM		
			50.46 ± 0.97 μM		
	Molecule 39	C6	0.5 μM	Caspases 3, Caspases 7, CK2, PARP	[141]
	Molecule 40	U-87MG	0.22 nM	PI3-p110α	[142]
	Molecule 41	HepG2	0.321 μM	c-Met	[143]
	Molecule 42	A-549	0.80 ± 0.12 μM	PI3K	[144]
	Molecule 43		0.29 ± 0.03 μM		
		MKN-45	6.49 ± 2.49 μM		
			0.80 ± 0.39 μM		
		H-460	9.59 ± 2.83 μM		
			0.59 ± 0.28 μM		
		U-87MG	8.66 ± 2.52 μM		
			0.34 ± 0.09 μM		
		HT-29	8.48 ± 3.21 μM		
			0.57 ± 0.08 μM		
		SMMC-7721	7.27 ± 2.97 μM		
			0.69 ± 0.40 μM		
**Indole containing heterocycles**	Molecule 44	U118MG	14.56 μM^#^	CHK1, GS3Kα, GS3Kβ, PDK1/AKT	[145]
		ANGM-CSS	7.73 μM^#^		
	Molecule 45	U-87MG	0.44 μM	PDK1, Aurora kinase A	[146]
	Molecule 46		0.008 μM		
	Molecule 47	U-87MG	112 nM	PI3K/PDK1/AKT	[147]
	Molecule 48	U-87MG	4.9 ± 0.5 μM	MDM2-CDK4	[148]
		U-251	8.6 ± 0.6 μM		
		T98-G	9.5 ± 0.7 μM		
	Molecule 49	A-549	2.2 μM	RAF/MEK/ERK, PI3K/PDK1/AKT	[149]
	Molecule 50		1.8 ± 0.8 μM		
		H460	0.3 μM		
			18.2 ± 0.7 μM		
**Acridone containing heterocycles**	Molecule 51	MCF-7	5.2 ± 1.2 μM	MARK4	[151]
	Molecule 52		6.3 ± 1.2 μM		
	Molecule 53		5.8 ± 1.4 μM		
		HepG-2	1.80 ± 0.04 μM		
			2.20 ± 0.05 μM		
			4.5 ± 0.52 μM		
**Triazine containing heterocycles**	Molecule 54	A-549	54–341 nM	PI3K	[152]
		D54	0.015–56.7 nM	MEK1	
	Molecule 55	U-87MG	0.23 μM	FAK	[153]
**Other heterocycles**	Molecule 56	GBM-95	27.1 μM	DYRK1A	[154]
		U87	28.7 μM		
	Molecule 57	DE3	3.3 ± 0.3 μM	DYRK1A	[155]
	Molecule 58		30 ± 9.5 μM		
	Molecule 59	U-251	5.82 μM	EGFR	[156]
	Molecule 60	U87EGFRvIII	0.5 μM	mTOR	[158]
	Molecule 61	M059K	40 μM	CK2, S/TK	[159]
	Molecule 62	V600E	ND	RAF/MEK/ERK	[160]
	Molecule 63	HT-29	118 nM	ERK1/2	[161]
	Molecule 64	NCI-60	0.71 µM	ERK2, JNK2	[162]
	Molecule 65	HEK293	8.9 ± 2.0 µM	MEK/ERK, PI3K/AKT	[163]
	Molecule 66		>100 µM		
		A549	21.6 ± 6.1 µM		
			>100 µM		
		A375	7.7 ± 1.1 µM		
			>100 µM		
		HL60	17.2 ± 6.6 µM		
			>100 µM		

Note: *EC_50_; ^#^GI_50_; ND-Not determined.

## Limitations and Future Perspective

In terms of treating GBM clinically, most of the currently available inhibitors have not proven successful. On the other hand, there are lack of precise targets. Many current targets including mTOR and PI3K, are targets of peripheral tumors, which results in comparatively significant toxicity and adverse effects. On the other hand, it is challenging for medications to penetrate the CNS and achieve a sufficient effective concentration because of the BBB’s limitations [164]. Kinase inhibitors comprise the bulk of small-molecule inhibitors used in the treatment of GBM. To obtain the appropriate pharmacological exposure, CNS drug candidates need to have optimum brain penetration in addition to high potency and selectivity against the chosen therapeutic target [165]. Since lipophilic substances are not soluble in water, developing derivatives is a crucial step in enabling molecules to penetrate the BBB by lipid-mediated diffusion, which requires high lipophilicity of the molecules. Aside from being a cutting-edge substitute for existing treatments for glioblastoma, heterocyclic compounds from both natural and synthetic derivatives serve as a preferred structure in the design of anticancer drugs [166]. Therefore, the use of heterocyclic compounds in the development of anti-tumor medicines appears promising. Most substances with a nonpolar moiety in their structure will make molecules more lipophilic and able to cross the blood-brain barrier. The active substances mostly target kinases, such as PI3K, DYRK, PDK1, CK2, c-Src, AKT/PKB, FAK and EGFR, in both *in vitro* and *in vivo* investigations. Additionally, many enzymes, biological functions and signaling paths are involved [167].

**Figure 11:**
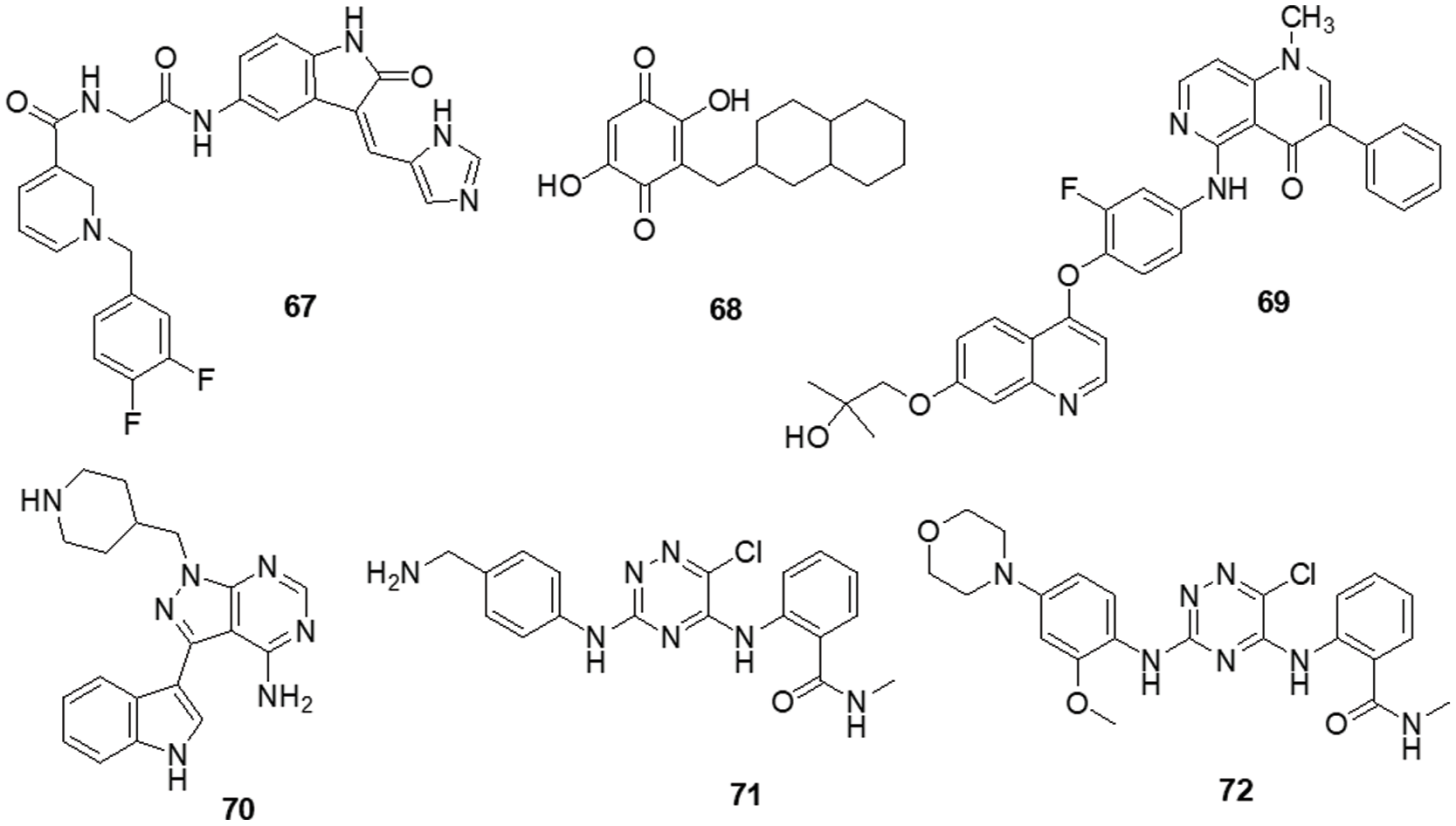
Chemical structures of novel derivatives against glioma.

The researchers discovered new, novel compounds that are heterocycles with various varieties. Heterocycles that include nonpolar groups are essential for the treatment of glioblastoma [168]. Because of its diverse effects on cellular activities, especially those related to cancer, indole and its derivatives have a special place in medicinal chemistry. Aurora kinase 1 and PKD1 (IC_50_ = 0.41 µM) were both inhibited by indole derivative **67** [147]. Benzoquinone derivative **68** has anticancer properties that effectively inhibit the growth of human glioma cells (U87MG) with an IC_50_ value of 23.6 µM. This finding demonstrates its ability to regulate cell cycle arrest and deactivate the NF-κB pathway [169]. Xu et al. [170] synthesized quinoline-based type II c-Met kinase inhibitors and they used a xenograft model to assess the inhibition of U-87MG glioma cells. With an IC_50_ value of 10.6 nM, compound **69** is the most effective type II c-Met kinase inhibitor that has been shown to reduce tumor cell proliferation by up to 93% in a glioblastoma xenograft model. Pyrimidine and its analogs are fundamental pharmacophores in the synthesis of several pharmaceuticals. A novel pyrazolo [3,4-d] pyrimidine-based derivative **70** was synthesized and its IC_50_ of 17–35 nM inhibitory action against PKD-dependent cortactin phosphorylation was found [171]. A newer series of heterocyclic compounds based on triazine and showing potential as antitumor agents has been reported. Compounds **71** and **72** showed strong anti-glioma activity with IC_50_s of 0.014 and 0.23 µm, respectively. Furthermore, compound **72** is the most effective inhibitor of focal adhesion kinase [153]. Additional alterations to these innovative substances may enhance their therapeutic uses. Drug research and development for GBM therapy will likely continue to focus on selective small-molecule agents with superior BBB permeability. Strategies for nano vectorization are a viable option that offers improved biocompatibility through passive targeting and increased permeability and retention impact. Enhancing active targeting is another way to increase the selectivity of cancer cells [172]. Researchers should also focus their attention on finding ways to combine small-molecule inhibitors with currently prescribed medications in a way that will help GBM patients by enhancing their mutual effects (Fig. 11).

## Conclusion

Drug resistance is one of the leading issues in cancer treatment. The most challenging aspect of the design and development of cancer treatment is dealing with the growth rate and recurrence of cancer. All these parameters must be considered in the drug discovery process. The target of the drug and mechanism of action plays an important role in drug cytotoxicity and lead to potent molecules. To address an issue with available treatment, it is important to study the signaling pathways of drugs. In this review, we have focused on the molecular pathways targeted by different heterocycles. The many types of heterocycles lead to changes in the pathways they target. The groups attached to the heterocycles, like aromatic or aliphatic and electron-donating or electron-withdrawing, differ in the potency of the molecules. The kinases are the most targeted enzymes in cancer. The different kinases are present in cells like RTKs, CDKs, LKs, etc. The activation of RTKs initiates many signaling cascades, resulting in various outcomes. Modify the expression of RTKs, their ligands and related proteins, focused on the MAPK, PI3K/AKT, JAK/STA and NF-κB downstream pathways. Subsequently, all the pathways, including RAS-RAF-MAPK, PI3K and AKT, are activated. The heterocycles that target receptor kinase pathways have been the main focus of this discussion. Pyrimidine, thiazole, imidazole, indole, acridone, triazine, etc., are all covered in this review targeting kinase pathways. The kinases are major contributors to cell metabolic pathways. Pathways like MGMT, IDH1/2 and others are yet to be studied for different heterocycles. The design of the lead compound shows great cytotoxicity and commercial value can be developed with various mechanistic pathways. The molecules targeting kinases might help target other pathways with some modification.

## Data Availability

The data cited in the review article is available on the internet on various platforms.
